# The relationships between ethnoracial identity, Aβ positivity, *APOE*ε4, and medial temporal lobe tau PET

**DOI:** 10.1002/alz.71226

**Published:** 2026-03-04

**Authors:** Koral V. Wheeler, Victoria R. Tennant, Noelle N. Lee, Maxwell W. Hand, Suchita Ganesan, Patrick Walsh, Meral Tubi, Jamie A. Terner, Brandon Hall, Marylan Davison, Arthur W. Toga, Sid O'Bryant, Alexandra L. Clark, Kristine Yaffe, Meredith N. Braskie, for the HABS‐HD Study Team, Meredith Braskie, Kevin King, James R Hall, Melissa Petersen, Raymond Palmer, Robert Barber, Yonggang Shi, Fan Zhang, Rajesh Nandy, Roderick McColl, David Mason, Bradley Christian, Nicole Phillips, Stephanie Large, Joe Lee, Badri Vardarajan, Monica Rivera Mindt, Amrita Cheema, Lisa Barnes, Mark Mapstone, Annie Cohen, Amy Kind, Ozioma Okonkwo, Raul Vintimilla, Zhengyang Zhou, Michael Donohue, Rema Raman, Matthew Borzage, Michelle Mielke, Beau Ances, Ganesh Babulal, Jorge Llibre-Guerra, Carl Hill, Rocky Vig

**Affiliations:** ^1^ Mark and Mary Stevens Neuroimaging and Informatics Institute Keck School of Medicine University of Southern California Los Angeles California USA; ^2^ University of North Texas Health Science Center Fort Worth Texas USA Fort Worth Texas USA; ^3^ Department of Psychology University of Texas at Austin Austin Texas USA; ^4^ Department of Psychiatry Neurology, and Epidemiology and Biostatistics University of California San Francisco School of Medicine San Francisco California USA

**Keywords:** amyloid‐beta positivity, *APOE*ε4, ethnoracial identity, learning and memory performance, medial temporal lobe, tau PET imaging

## Abstract

**INTRODUCTION:**

Clarifying relationships between amyloid, tau, and cognition is crucial to understanding dementia risk, but has been mainly performed in non‐Hispanic White (NHW) participants. It is unknown whether findings are generalizable to other ethnoracial groups.

**METHODS:**

We evaluated relationships between amyloid‐β (Aβ) positivity, apolipoprotein E allele (*APOE)* ε4, tau‐positron emission tomography (PET) ^18^F‐PI‐2620, and cognitive performance in 1181 cognitively unimpaired (451 NHW, 353 Hispanic, and 377 Black) and 383 mild cognitively impaired (85 NHW, 129 Hispanic, and 169 Black) participants from the Health and Aging Brain Study‐Health Disparities.

**RESULTS:**

Black (*β* = 0.28, *p* < 0.001) and Hispanic (*β* = 0.34, *p* < 0.001) participants had higher medial temporal lobe (MTL) tau than NHW participants; however, findings were attenuated when accounting for choroid plexus off‐target binding. Hispanic participants showed higher tau in lateral temporal regions compared to NHW and Black participants; however, reducing meningeal off‐target binding through erosion demonstrated similar lateral temporal tau across groups.

**DISCUSSION:**

Factors other than amyloid and tau may impact cognition in Black participants. PI2620 off‐target ethnoracial differences should be investigated.

## Highlights

- Hispanic and Black participants showed higher medial temporal lobe (MTL) tau levels than non‐Hispanic White (NHW) participants.
- The relationship between amyloid‐β (Aβ), MTL tau, and cognition differed in the Black cohort.
- Choroid plexus off‐target binding partially contributed to MTL tau signal.
- Lateral temporal tau was the same across groups upon removing meningeal binding.

## 1 BACKGROUND

Alzheimer's disease (AD) is characterized by the aggregation of amyloid‐β (Aβ) plaques and neurofibrillary tau tangles that disrupt neuronal functioning and lead to cognitive decline. Non‐Hispanic Black (henceforth Black) and Hispanic/Latino(a) (henceforth Hispanic) older adults are at increased risk for AD and related dementias (ADRD) compared to non‐Hispanic White (henceforth NHW) older adults.^1^, ^2^, ^3^ Black and Hispanic older adults are more likely to be exposed to environmental and socioeconomic stressors across the life course, and experience higher rates of cardiometabolic conditions relative to NHW older adults. Greater exposure to these risk factors may contribute to disparities in brain aging and AD risk for racial/ethnically minoritized older adults.^4^, ^5^, ^6^, ^7^ Despite this, AD research overwhelmingly lacks diverse and representative study samples that vary in comorbidities,^8^, ^9^, ^10^, ^11^ which limits generalizability of current AD research models. The lack of ethnically diverse research cohorts that resemble the general and at‐risk aging populations impedes the field's understanding of AD biomarkers, precision medicine diagnostics, and treatments.^10^

Although tau is closely correlated with cognition, only a few studies have investigated tau positron emission tomography (PET) in ethnically diverse cohorts, with divergent findings.^12^, ^13^, ^14^ These mixed findings may be related to the small samples of Black participants, differences in the evaluated composite regions of interest, and PET off‐target binding.^12^, ^13^, ^14^ Our lab recently found that a medial temporal lobe (MTL) tau cut‐point differentiated cognitively unimpaired (CU) from cognitively impaired Hispanic and NHW participants but not Black participants in the Health and Aging Brain Study‐Health Disparities (HABS‐HD) cohort, raising questions as to whether other factors are more correlated with cognitive impairment in Black participants than MTL tau.^15^ Our current study seeks to further investigate the associations between MTL tau and cognitive performance across ethnoracial groups during preclinical stages by analyzing the moderating effects of Aβ and apolipoprotein E allele (*APOE*ε4) positivity within CU and mild cognitively impaired (MCI) participants.

*APOE*ε4 is the strongest genetic AD risk factor, though this association has been most consistently found in NHW adults.^16^ *APOE*ε4 carriership is associated with higher rates of MTL tau accumulation, especially in Aβ‐positive participants,^17^ and a stronger relationship between MTL tau and poorer memory performance.^18^ However, the effect of *APOE*ε4 on AD risk varies by race/ethnicity, with some reports of Hispanic and Black participants showing weak or mixed associations between *APOE*ε4 and cognitive impairment compared to effects in NHW participants.^16^, ^19^, ^20^, ^21^, ^22^ One study found no association between *APOE*ε4 positivity and MTL tau PET in Black participants; however, the sample was small.^23^ Little is known about the relationships between *APOE*ε4 and tau PET, the effect of *APOE*ε4 on the relationship between amyloid and tau PET, or the impact of *APOE*ε4 on tau PET and cognitive performance among Hispanic or Black participants.

In an HABS‐HD sample of NHW, Hispanic, and Black participants without dementia, we assessed MTL tau relationships within the overall cohort and across ethnoracial groups. We investigated whether ethnoracial group moderated the associations between (1) MTL tau and Aβ‐PET positivity, a measure of pathological brain amyloid‐beta accumulation, and (2) MTL tau PET and cognitive performance. To further explore our findings, we investigated whether Aβ positivity interacted with MTL tau on memory performance. We assessed whether ethnoracial group moderated the associations between *APOE*ε4 and MTL tau. We also tested whether *APOE*ε4 moderated the associations between MTL tau and Aβ positivity as well as cognitive performance across the ethnoracial groups. To evaluate whether these relationships extended to cortical regions outside of the MTL, we tested the same associations in distinct lateral temporal regions, and composite regions of the posterior cingulate and lateral parietal cortices.

We used a second‐generation tau PET radiotracer, ^18^F‐PI‐2620 (PI‐2620) that has shown high selectivity for tau aggregates^24^, ^25^, ^26^ and low off‐target binding to monoamine oxidase compared to ^18^F‐flortaucipir.^27^ We performed sensitivity analyses to (1) evaluate whether off‐target PET binding from the choroid plexus adjacent to the hippocampus affects MTL PET signal and (2) assess whether meningeal off‐target binding impacts lateral temporal signal.

## 2 METHODS

Our study is a cross‐sectional analysis of data collected from the ongoing longitudinal HABS‐HD study which was approved at the University of North Texas Health Science Center (UNT HSC) and provides publicly available data through the UNT HSC's Institute for Translational Research. The primary objective of HABS‐HD is to examine associations between the onset and progression of ADRD, AD biomarkers, and relationships between AD and health, socioeconomic, environmental, and genetic factors in a racially/ethnically diverse cohort. An emphasis is placed on evaluating associations between health disparities and ADRD prevalence in NHW, Black, and Hispanic adults aged 30–90, although our current study includes only individuals who are aged 50 and older. An in‐depth description of the participant recruitment and HABS‐HD study methods has been previously published.^28^ Participants provide blood samples, undergo neuroimaging scans, and complete clinical interviews and functional and neuropsychological assessments. Clinical information regarding history of hypertension, diabetes, dyslipidemia, and cardiovascular disease was gathered through medical interview and examination at research visits. Diagnoses were confirmed or made by a licensed practitioner (either a physician or nurse practitioner) after clinical evaluation.

**RESEARCH IN CONTEXT**

1. **Systematic review**: The authors reviewed literature on PubMed. Most publications have investigated cognitive, amyloid, and tau positron emission tomography (PET) biomarkers by predominantly including highly‐educated NHW participants with low cardiometabolic risk. The ability to accurately generalize findings to ethnoracially‐diverse participants have been restricted.
2. **Interpretation**: Cognitively unimpaired (CU) Hispanic and Black participants show higher medial temporal lobe (MTL) tau levels than CU NHW participants even after controlling for choroid plexus off‐target binding. Amyloid‐β (Aβ) ‐positivity moderated MTL tau and cognitive relationships in NHW and Hispanic but not Black participants, suggesting that factors other than tau and amyloid may more strongly predict impairment in Black participants. Hispanic participants showed higher lateral temporal tau levels including in CU Aβ− cohorts; however, after reducing off‐target meningeal signal, lateral temporal tau levels were comparable across ethnoracial groups.
3. **Future directions**: Additional research is needed to evaluate the neurochemical and neuroanatomical contributors to ethnoracial differences in PET off‐target binding.

### 2.1 Participants

We examined 1181 CU and 383 MCI participants who participated in the HABS‐HD study between January 2020 and August 2024 and had usable tau and amyloid PET scans and clinical/cognitive data collected within 1 year of each other. Out of 2229 participants who had tau PET acquired during the time of the analysis, we excluded participants based on usability of tau PET data for the following reasons: 1 participant due to an acquisition error, 1 participant due to a scanner artifact, 2 participants due to anatomical abnormalities, 3 participants because of excessive motion, 7 participants with excessive signal spillover into the reference region, 11 participants because of failed reference region segmentations based on our quality control assessment for tau PET, and 37 participants because of failed MTL segmentations based on our quality control assessment. Of the remaining participants, 1661 participants were diagnosed as CU or MCI at baseline who had available clinical and cognitive data. Out of these 1661 participants, 1636 participants had amyloid PET scans that were acquired and analyzed. Participants were then eliminated based on the usability of amyloid PET data as follows: 1 participant due to an acquisition error, 2 participants due to a field of view error, 4 participants due to excessive motion, 6 participants due to anatomical abnormalities, 29 participants due to failed region of interest (ROI) segmentations based on our quality control assessment for amyloid PET, and 26 participants due to failed reference region segmentations based on our quality control assessment. Additionally, 4 participants were excluded because the time between clinical and amyloid, clinical and tau, or amyloid and tau PET visits exceeded 1 year. Our final sample included 451 NHW, 353 Hispanic, and 377 Black CU participants and 85 NHW, 129 Hispanic, and 169 Black MCI participants. Out of the Hispanic participants, 418 identified as Mexican American, 7 identified as Puerto Rican, 3 identified as Cuban, and 54 identified as another Hispanic subgroup. Table 1 describes sample demographics.

**TABLE 1:** Demographics of study sample.

Characteristic	Non‐Hispanic White	Hispanic	Black	*p*‐value
Total participants	536	482	546	
**Hispanic subgroups**				
Mexican American	–	418	–	
Puerto Rican	–	7	–	
Cuban		3		
**Other subgroups**		54		
CU	451 (84%)	353 (73%)	377 (69%)	<0.001
MCI	85 (16%)	129 (27%)	169 (31%)	<0.001
**Aβ (*n*) %**				
CU				<0.001
Aβ positivity	105 (23%)	42 (12%)	48 (13%)	
Aβ negativity	346 (77%)	311 (88%)	329 (87%)	
MCI				<0.001
Aβ positivity	31 (36%)	25 (19%)	24 (14%)	
Aβ negativity	54 (64%)	104 (81%)	145 (86%)	
**Age in years—mean (SD)**				
CU	69 (8)	63 (8)	62 (7)	<0.001
MCI	70 (9)	65 (9)	62 (7)	<0.001
**Sex (*n*) %**				
CU				0.004
Female	274 (61%)	249 (71%)	263 (70%)	
Male	177 (39%)	104 (29%)	114 (30%)	
MCI				0.073
Female	44 (52%)	75(58%)	76 (45%)	
Male	41 (48%)	54(42%)	93 (55%)	
***APOE*ε4 (*n*) %**				
CU				<0.001
Positive	122 (27%)	58 (16%)	112 (30%)	
Negative	298 (66%)	257 (73%)	184 (49%)	
Missing	31 (7%)	38 (11%)	81 (21%)	
MCI				0.008
Positive	25 (29%)	27 (21%)	58 (34%)	
Negative	51 (60%)	89 (69%)	81 (48%)	
Missing	9 (11%)	13 (10%)	30 (18%)	
**Cardiovascular disease (*n*) %**				
CU				0.542
Yes	36 (8%)	22 (6%)	24 (6%)	
No	415 (92%)	331 (94%)	353 (94%)	
MCI				0.093
Yes	11 (13%)	6 (5%)	16 (9%)	
No	74 (87%)	123 (95%)	153 (91%)	
**Hypertension (*n*) %**				
CU				<0.001
Yes	265 (59%)	223 (63%)	307 (81%)	
No	186 (41%)	130 (37%)	70 (19%)	
MCI				0.097
Yes	55 (65%)	85 (66%)	127 (75%)	
No	30 (35%)	44 (34%)	41 (24%)	
Missing	0	0	1 (1%)	
**Dyslipidemia (*n*) %**				
CU				<0.033
Yes	313 (69%)	258 (73%)	242 (64%)	
No	138 (31%)	95 (27%)	135 (36%)	
MCI				0.007
Yes	57 (67%)	98 (76%)	99 (59%)	
No	28 (33%)	31 (24%)	70 (41%)	
**Diabetes (*n*) %**				
CU				<0.001
Yes	68 (15%)	127 (36%)	114 (30%)	
No	371 (82%)	218 (62%)	242 (64%)	
Missing	12 (3%)	8 (2%)	21 (6%)	
MCI				0.054
Yes	23 (27%)	55 (43%)	54 (32%)	
No	60 (71%)	72 (56%)	107 (63%)	
Missing	2 (2%)	2 (1%)	8 (5%)	
**Education in years – mean (SD)**				
CU	16 (2)	12 (4)	15 (3)	<0.001
MCI	15 (3)	11 (4)	14 (2)	<0.001
**Income in US dollars – mean (SD)**				
CU	100,238 (99,303)	52,944 (47,583)	85,441 (101,054)	<0.001
MCI	71,958 (68,526)	46, 348 (52,112)	55,293 (59,124)	0.013

Abbreviations: Aβ, amyloid‐β; *APOE*ε4, apolipoprotein E allele; CU, cognitively unimpaired; MCI, mild cognitively impaired.

*p* values demonstrate differences across ethnoracial groups.

### 2.2 Cognitive diagnoses

Clinical interviews, Clinical Dementia Rating Scale (CDR) Sum of Boxes, and cognitive test performance were used to determine cognitive diagnoses. CU participants had a CDR Sum of Boxes score of 0 and cognitive test scores better than 1.5 standard deviations (SDs) below the mean while MCI participants had a CDR Sum of Boxes score between 0.5 and 2.0, and at least one cognitive test score fell at or below 1.5 SDs beneath z‐adjusted norms. Dementia patients were not included in the current study due to our interest in understanding early AD marker associations. Age, race/ethnicity, sex, and years of education were self‐reported at clinical visits.

### 2.3 *APOE*ε4 genotyping

*APOE*ε4 genotyping was performed using TaqMan Genotyping Kits and variants were checked for Hardy Weinberg equilibrium. Participants who carried at least one *APOE*ε4 allele were categorized as *APOE*ε4 positive.

### 2.4 Neuropsychological evaluation

All HABS‐HD study interviews and testing were conducted in either Spanish or English depending on participant preference. Participants completed a full neuropsychological battery. We focused on MTL‐dependent cognitive tests that measure episodic memory through immediate and delayed recall performance: Spanish English Verbal Learning Test (SEVLT) Trials 1–5 total, SEVLT 30‐min Delayed Recall total, and the Wechsler Memory Scale, third edition (WMS‐III) Logical Memory immediate and delayed story recall total. We calculated a z‐score memory composite that averaged the z‐scores for each of these tests. A Cronbach's alpha value of 0.90 was calculated indicating that all items reliably measured the memory construct.^29^

### 2.5 Neuroimaging

All neuroimaging scans were acquired at the UNT HSC and stored in the Imaging & Data Archive (IDA) maintained by the University of Southern California. Scans were downloaded from the IDA and processed at the University of Southern California.

#### 2.5.1 MRI acquisition and processing

Participants were scanned on either a Siemens Magnetom Skyra 3T whole‐body MRI or Siemens Magnetom Vida 3T MRI located at the Institute for Translational Research at the University of North Texas. Within our sample, 51 participants were scanned on the Skyra, and 1513 participants were scanned on the Vida. For each participant, we acquired a T1‐weighted whole brain volumetric spoiled magnetization‐prepared rapid gradient (MPRAGE) scan (Skyra: repetition time [TR] = 2300 ms; echo time [TE] = 2.93 ms, 1.1 × 1.1 × 1.2 mm voxels; Vida: TR = 2300 ms, TE = 2.98 ms, 1.0 × 1.0 × 1.0 mm voxels; flip angle = 9 for Skyra and Vida).

We downloaded unprocessed MRI scans from the Laboratory of NeuroImaging IDA website. MRI images were bias corrected using the Advanced Normalization Tools (ANTS) with parameters of bspline‐fitting = [200], shrink factor = 1, and convergence = [50 × 50 × 45 × 40, 0]. The bias‐corrected images served as input for FreeSurfer version 5.3.0, which includes an additional bias‐correction step. FreeSurfer was used to parcellate and segment all cortical and subcortical regions of interest of the T1‐weighted volume scans.

#### 2.5.2 Tau PET acquisition and processing

Participants underwent tau PET imaging on one of two identical Siemens Biograph Vision 450 scanners located at the Institute for Translational Research at UNT. Participants were injected with a 5 mCi bolus ± 10% of PI‐2620. A CT scan was acquired for attenuation correction. Forty‐five to 75 min postinjection, a dynamic emission scan was acquired (six frames of 5 minutes each). The PET images were reconstructed after the 30‐min scan. Reconstruction was iterative (eight iterations; five subsets) with time of flight on, a grid of 440 × 440 × 119, zoom of 2, and an all‐pass filter on.

We downloaded unprocessed, reconstructed tau PET scans from the Laboratory of NeuroImaging IDA website. To correct for participant motion, all frames were co‐registered to the first frame using statistical parametric mapping (SPM) 12's reslice and realign tool followed by an averaging of motion‐corrected frames to create one 3D PET image. FreeSurfer version 5.3 was used to segment MRI T1‐weighted scans. Our study's primary focus was our standard MTL ROI (entorhinal cortex, hippocampus, and amygdala) as the site of early tau accumulation, including in CU individuals.^30^ We performed a standardized uptake value ratio (SUVR) analysis using inferior cerebellar gray matter as a reference region, consistent with prior work.^24^, ^31^ Based on our reference region testing for PI‐2620,^32^ and as detailed in our prior work,^15^ we used the SUIT cerebellar atlas in Montreal Neurological Institute (MNI) space to exclude all cerebellar regions superior to the left and right Crus I lobule. The inferior cerebellum binary mask in MNI space was non‐linearly aligned to native T1 space for each participant and multiplied by the individual participants’ FreeSurfer‐derived cerebellar gray matter masks in T1 native space to derive each participant's native‐MR‐space reference region. After PET and T1‐weighted MR images were linearly co‐registered, all regions of interest were transformed to native PET space, where the SUVR analyses took place. During the quality control process, a final determination was made as to whether signal spillover was present in the inferior cerebellum and if there were more than four rows of voxels of contamination in the reference region, the scan was excluded. MTL SUVRs were calculated by dividing the mean raw MTL tau PET uptake values by the mean raw reference region PET uptake values to derive a mean SUVR for MTL tau.

Black participants may demonstrate higher tau PET signal unrelated to tau accumulation compared to NHW participants due to off‐target binding in the choroid plexus.^13^ We tested whether neighboring off‐target signal affects the MTL tau PET levels across the ethnoracial groups. To do this, we extracted the Freesurfer‐derived choroid plexus segmentation, co‐registered it to native PET space and obtained the mean tau PET uptake values. We performed sensitivity testing by re‐evaluating all main statistical analyses while adjusting for choroid plexus SUVR.

We also assessed whether the results we found in the MTL would be reflected in more advanced stage tau regions of the brain. We tested these associations in separate lateral temporal regions including the fusiform, inferior temporal, middle temporal, and parahippocampal gyrus. We performed a sensitivity analysis to test whether off‐target signal from the meninges contributed to ethnoracial differences in lateral temporal tau by creating eroded lateral temporal regions for increased accuracy of SUVR calculations. These were created by eroding a full brain mask using FSL erode kernel sphere 3. The full brain mask was then multiplied by Freesurfer‐derived fusiform, inferior temporal, middle temporal, and parahippocampal masks, resulting in masks that excluded the voxels on the pial surface that were contiguous with the cerebral spinal fluid or meninges.

Lastly, we tested whether ethnoracial differences were observed in more advanced cortical regions, according to Braak and Braak staging, by using composite ROIs of the lateral parietal (a combination of the inferior parietal, superior parietal, and supramarginal) and posterior cingulate cortex regions (a combination of the isthmus cingulate and posterior cingulate). The same erosion method above was applied to the lateral parietal mask to create an eroded lateral parietal region to reduce inclusion of meningeal off‐target binding. In prior work from our lab, this eroded lateral parietal region removed signal that was likely adding statistical noise from off‐target contamination and modestly strengthened relationships between our lateral parietal ROI and age, cognitive status, and MTL tau signal.^33^ Same as for the MTL SUVRs, SUVRs for the distinct lateral temporal regions, lateral parietal, and posterior cingulate were calculated by dividing each of the mean raw tau PET regional signal by the mean raw reference region PET signal.

#### 2.5.3 Amyloid PET acquisition and processing

Participants underwent amyloid PET imaging using the Siemens Biograph Vision 450 whole‐body PET/CT scanners that were also used for the tau PET scans, using the ADNI3 protocol for florbetaben (Neuraceq) scans. Per the scanning protocol, participants were injected with an 8.1 mCi bolus ± 10% florbetaben. A low‐dose CT scan was acquired for attenuation correction. Ninety minutes after bolus injection, a dynamic emission scan was acquired (four 5‐min frames). Reconstruction was performed using the same parameters as listed for the Siemens Biograph Vision 450 scanner above for PI‐2620.

We downloaded unprocessed, reconstructed amyloid PET scans from the Laboratory of NeuroImaging IDA website. Scans were first motion corrected by co‐registering each frame in a scan to the participant's first frame using SPM 12′s re‐slice and realign functions. Frames were then averaged and consistent with the ADNI3 analysis protocol for florbetaben, scans were resampled to a voxel size of 1.5 mm and smoothed with a kernel of 6.5 mm in‐plane and 5.5 mm through‐plane to an apparent resolution of ∼8 mm. FreeSurfer version 5.3.0 was used to segment each participant's MRI T1‐weighted scan into frontal, lateral parietal, anterior/posterior cingulate, and lateral temporal ROIs as described in the ADNI3 protocols for florbetaben^34^ and florbetapir.^35^ We also used FreeSurfer to delineate the whole cerebellum reference region for florbetaben. Each participant's PET scan was linearly co‐registered to the T1‐weighted MR images (6 degrees of freedom) using a mutual information cost function. The inverse coregistration matrices were applied to the ROIs and reference regions to bring them into native PET image space, where our analyses were performed. We calculated a global summary of amyloid PET SUVR by calculating the mean of the frontal, lateral parietal, anterior/posterior cingulate, and lateral temporal SUVRs. For the purposes of this study, participants were categorized as Aβ+ (global SUVR at least 1.08) or Aβ− (below 1.08) consistent with ADNI3 guidelines for florbetaben.^36^ Since the Aβ positivity rates of the study were at 18%, we tested that the ADNI3 amyloid positivity cutoff was not too high to use with this cohort by performing a Gaussian mixture model analysis. Our results showed that the estimated cutoff value was close to the ADNI3 cutoff value indicating that the use of this cutoff value was appropriate for our cohort. Methods for this process are specified in the Supporting Information S1 section. Density curves of global amyloid SUVRs are presented in Figure S1.

### 2.6 Statistical analysis

All statistical analyses and data visualizations were conducted using R studio version 2023.06.2+561. To reduce the influence of data outliers, robust regression models were used to investigate associations involving ethnoracial group, Aβ positivity, mean MTL tau SUVR, *APOE*ε4, and memory performance. Robust regressions were also used to assess the separate relationships between ethnoracial group, Aβ positivity, cognitive performance and mean lateral temporal regions, the lateral parietal composite and posterior cingulate composite SUVRs. Covariates included were age, sex, years of education, and Aβ positivity. We performed false discovery rate (FDR) adjustments for multiple comparisons when necessary, with the statistical significance after correction at *p* < 0.05. Robust regression results are presented as standardized beta estimates, confidence intervals, and *p* values.

## 3 RESULTS

### 3.1 Sample characteristics

As presented in Table 1, we used analyses of variance (ANOVAs) to compare differences among ethnoracial groups in mean age, education, and annual household income. Chi‐squared tests of independence were used to assess differences in cognitive diagnosis, Aβ positivity, sex, *APOE*ε4 positivity, cardiovascular disease, hypertension, dyslipidemia, and type 2 diabetes diagnoses across ethnoracial groups.

To ensure that results were not confounded by group differences in disease progression, we performed 1‐to‐1 matching of NHW and Black pairs and NHW and Hispanic pairs on age (±3‐year difference), gender, education in years, and cognitive diagnosis. We re‐performed all main analyses using these matched groups. Methods and results for these analyses are separately presented in the Supporting Information S2 section.

### 3.2 MTL tau levels differed across racial/ethnic groups

We evaluated MTL tau levels (using our standard MTL regions: entorhinal cortex, hippocampus, and amygdala) among ethnoracial groups using robust regression analyses controlling for age, sex, education, and Aβ positivity.

In the combined CU and MCI cohort, Hispanic (*β* = 0.34, 95% CI [0.23, 0.44], *p* < 0.001) and Black (*β* = 0.28, 95% CI [0.19, 0.38], *p* < 0.001) participants had higher MTL tau PET signal than NHW participants. Stratified by cognitive diagnosis, CU Hispanic (*β* = 0.35, 95% CI [0.24,0.46], *p* < 0.001) and Black (*β* = 0.32, 95% CI [0.21,0.43], *p* < 0.001) participants also had higher MTL tau PET SUVR than CU NHW participants but this difference was not observed in MCI participants (0.12 < *β* < 0.21, *p* > 0.159) (Table S1). Cognitive diagnosis significantly moderated the relationship between MTL tau and ethnoracial group in which NHW participants showed significantly higher levels of MTL tau associated with MCI compared to Black participants (*β* = −0.31, 95% CI [−0.57, −0.06], *p *= 0.017).

**FIGURE 1:**
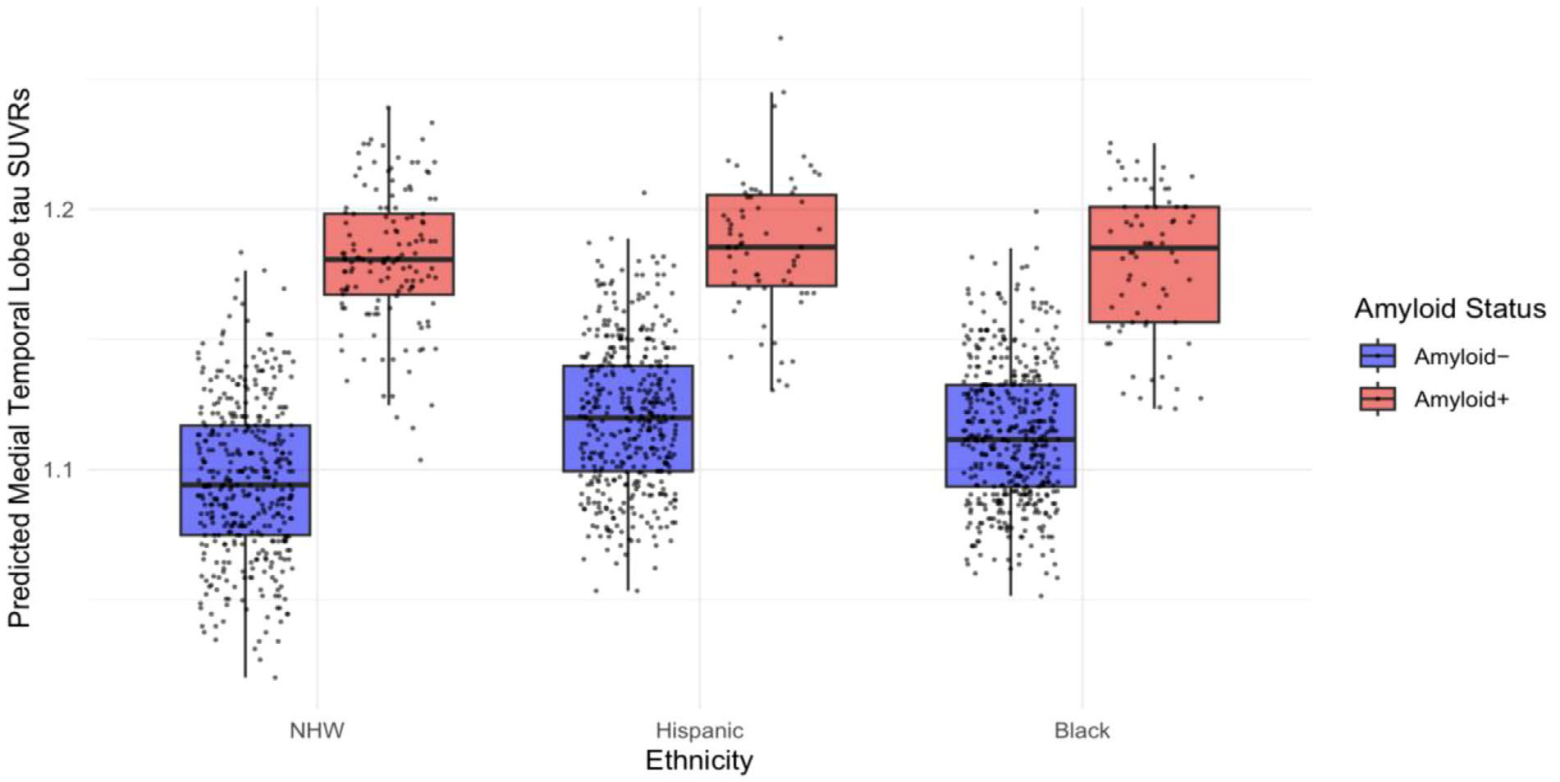
Predicted tau MTL levels by ethnoracial group and Aβ positivity. Boxplots of predicted MTL tau SUVRs by ethnoracial group and Aβ positivity status show that in Aβ− participants, Black (*β* = 0.28, 95% CI [0.18, 0.38], *p* < 0.001) and Hispanic (*β* = 0.34, 95% CI [0.24, 0.45], *p* < 0.001) participants showed higher MTL tau levels than NHW participants while all Aβ+ ethnoracial groups showed similar MTL tau levels (0.26 < *β* < 0.06 *p* > 0.185). Aβ, amyloid‐β; CI, confidence interval; MTL, medial temporal lobe; NHW, non‐Hispanic White; SUVR, standardized uptake value ratio.

We then investigated Aβ associations with MTL tau levels across ethnoracial groups. In CU participants, ethnoracial group did not moderate the association between Aβ positivity and MTL tau between either CU NHW and Hispanic participants (*β* = −0.13, 95% CI [−0.54, 0.28], *p *= 0.530) or CU NHW and Black participants (*β* = 0.05, 95% CI [−0.39, 0.49], *p *= 0.834) (Table S2). Likewise, within the MCI cohort, ethnoracial group did not moderate the associations between MTL tau and Aβ positivity between either NHW and Black participants (*β* = −0.88, 95% CI [−1.88, 0.12], *p *= 0.084) or NHW and Hispanic participants (*β* = −0.53, 95% CI [−1.40, 0.35], *p *= 0.239). Table 2 provides a summary of mean MTL tau SUVRs across the groups. Figure 1 illustrates boxplots of the predicted MTL tau levels by ethnoracial group and Aβ positivity status controlling for age, sex, and education.

**TABLE 2:** MTL tau SUVRs across ethnoracial groups by cognitive diagnosis and Aβ+ positivity.

Cognitive diagnosis	Aβ+ status	Hispanic	Black	NHW
Total CU–mean (SD)		1.129 (0.103)	1.129 (0.120)	1.122 (0.136)
	Aβ−	1.125^*^ (0.097)	1.117 (0.107)	1.103 (0.122)
	Aβ+	1.152 (0.140)	1.209 (0.164)	1.188 (0.157)
Total MCI–mean (SD)		1.159 (0.162)	1.134 (0.146)	1.182 (0.178)
	Aβ−	1.122 (0.115)	1.116 (0.111)	1.100 (0.093)
	Aβ+	1.315 (0.227)	1.240 (0.255)	1.325 (0.201)

Table of mean and standard deviations of MTL tau SUVRs by cognitive diagnosis and Aβ positivity across ethnoracial groups.

Abbreviations: Aβ, amyloid‐β; CU, cognitively unimpaired; MCI, mild cognitively impaired; MTL, medial temporal lobe; NHW, non‐Hispanic White; SUVR, standardized uptake value ratio.

* Indicates a statistically different MTL tau mean compared to the NHW group.

### 3.3 Ethnoracial group did not moderate the associations between MTL tau and memory scores

We evaluated the associations between MTL tau PET and a memory composite score across the entire cohort by assessing whether ethnoracial group moderated the associations between MTL tau PET and memory while covarying for age, sex, education, and Aβ‐positivity. Within the whole cohort of MCI and CU participants, higher MTL tau was associated with a lower composite learning and memory score (*β* = −0.12, 95% CI [−0.18, −0.06], *p* < 0.001). To evaluate which aspects of learning and memory drove this significant association between memory and MTL tau, we further tested tau relationships with each individual cognitive test used to create the composite score (the SEVLT immediate and delayed free recall tests, and the Logical Memory (A+B) immediate and delayed recall tests). For all individual tests within the learning and memory composite score, poorer performance was associated with higher MTL tau (SEVLT immediate recall (*β* = −0.11, 95% CI [−0.17, −0.05], *p* < 0.001), SEVLT delayed recall (*β* = −0.14, 95% CI [−0.21, −0.07], *p* < 0.001), Logical Memory Story (A+B) immediate recall (*β* = −0.11, 95% CI [−0.16, −0.05], *p* < 0.001), and Logical Memory Story (A+B) delayed recall (*β* = −0.13, 95% CI [−0.19, −0.06], *p* < 0.001)).

We further assessed whether ethnoracial identity moderated the relationship between tau and cognitive performance. We found that no interaction effect of ethnoracial identity was present between MTL tau and memory composite when comparing NHW and Black participants (*β* = 0.12, 95% CI [−0.01, 0.26], *p *= 0.072), Black and Hispanic participants (*β* = 0.08, 95% CI [−0.03, 0.19], *p *= 0.171), or between NHW and Hispanic participants (*β* = 0.04,95%CI [−0.10, 0.19], *p *= 0.538). Results of individual tests among each ethnoracial group are presented in Table S3.

### 3.4 Aβ positivity moderated the associations between cognitive performance and MTL tau within NHW and Hispanic groups but not Black participants

**FIGURE 2:**
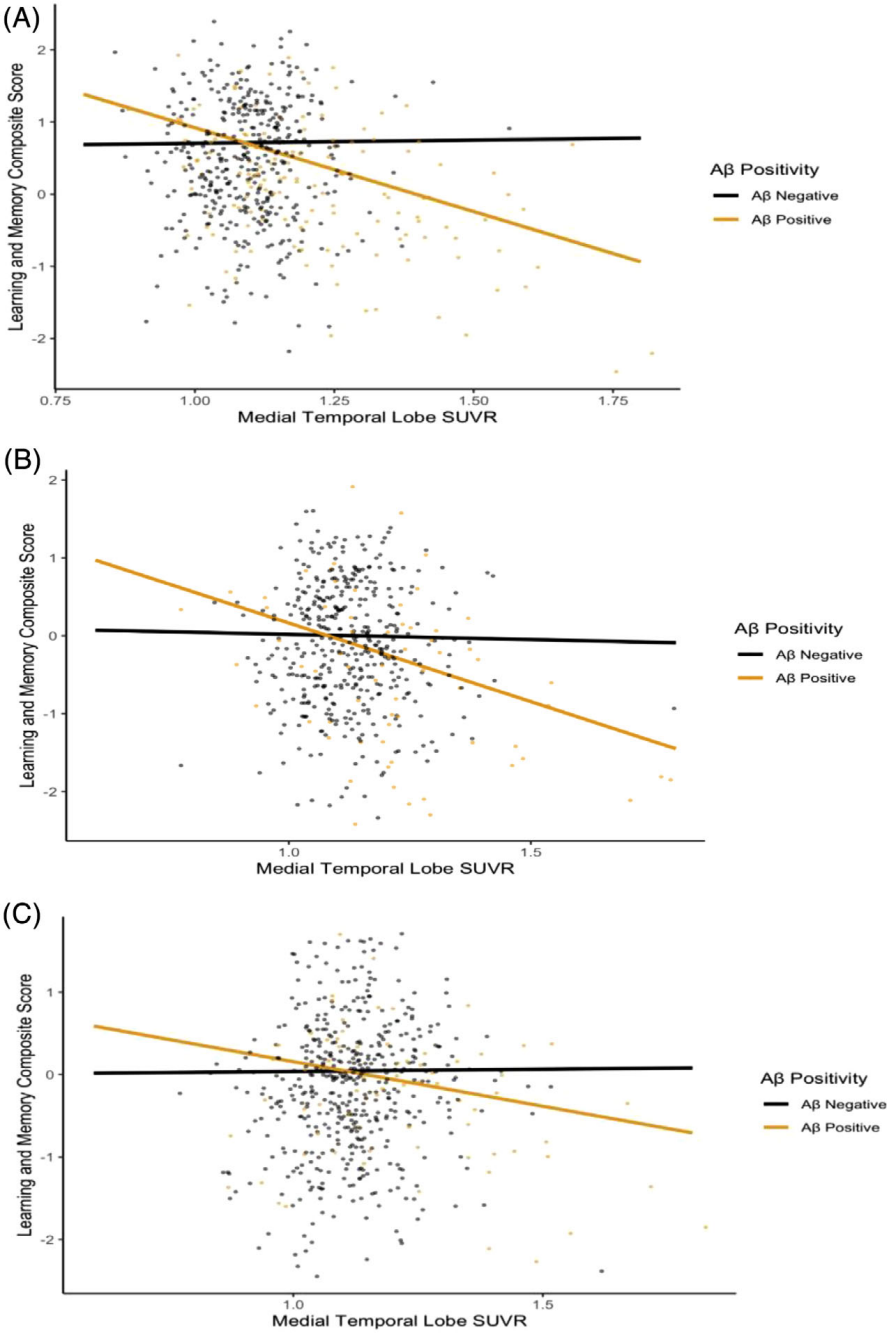
Relationships between Aβ positivity, MTL tau PET SUVR, and learning and memory composite score across ethnoracial groups in CU and MCI older adults. (A) Aβ positivity moderated the association between MTL tau SUVR and the learning and memory composite score in NHW participants (*β* = −0.31, 95% CI [−0.42, −0.19], *p* < 0.001). One participant was excluded from the figure (but not the analysis) for improved visualization since their MTL SUVR fell outside of the plotted *x*‐axis range. (B) Aβ positivity moderated the association between MTL tau SUVR and the learning and memory composite score in Hispanic participants (*β* = −0.25, 95% CI [−0.39, −0.10], *p* < 0.001). (C) Aβ positivity trended toward moderating the association between MTL tau SUVR and the learning and memory composite score in Black participants (*β* = −0.15, 95% CI [−0.31, 0.01], *p *= 0.067). Aβ, amyloid‐β; CI, confidence interval; CU, cognitively unimpaired; MCI, mild cognitive impairment; MTL, medial temporal lobe; NHW, non‐Hispanic White; PET, positron emission tomography; SUVR, standardized uptake value ratio.

We further explored the relationship between MTL tau and cognitive performance across ethnoracial groups by evaluating the potential interaction of Aβ positivity. Previous literature has indicated that Aβ positivity is associated with greater levels of tau within the MTL, which subsequently spreads to other neocortical regions and contributes to cognitive impairment.^37^ We tested whether Aβ positivity moderated the relationship between MTL tau and cognitive performance within each ethnoracial group and covaried for age, education, and sex. There was a significant interaction effect of Aβ positivity on the association between MTL tau and cognitive performance in both NHW participants (Figure 2A) (memory composite score (*β* = −0.31, 95% CI [−0.42,−0.19], *p* < 0.001)) and Hispanic participants (memory composite score (*β* = −0.25, 95% CI [−0.39,−0.10], *p* < 0.001) (Figure 2B). However, Aβ positivity did not moderate the associations between MTL tau and the composite memory score in Black participants (*β* = −0.15, 95% CI [−0.31, 0.01], *p *= 0.067) (Figure 2C). Table 3 summarizes these findings.

**TABLE 3 alz71226-tbl-0003:** (A) Associations between MTL tau and memory performance across ethnoracial groups by Aβ positivity, (B) interaction between Aβ positivity and MTL tau on memory performance, and (C) interaction between ethnoracial group and MTL tau on memory performance.

	NHW participants			Hispanic participants			Black participants		
Group	*β*	95% CI	*p*‐value	*β*	95% CI	*p*‐value	*β*	95% CI	*p*‐value
**(A)**									
Full cohort	−0.13	−0.24, −0.01	0.032	−0.12	−0.19, −0.04	0.005	−0.04	−0.12, 0.04	0.341
Aβ negative	−0.004	−0.06, 0.05	0.892	−0.03	−0.13, 0.08	0.645	0.01	−0.08, 0.11	0.764
Aβ positive	−0.30	−0.42, −0.19	<0.001	−0.23	−0.33, −0.13	<0.001	−0.18	−0.33, −0.03	0.019
**(B)**									
Aβ positivity interaction	−0.31	−0.42, −0.19	<0.001	−0.25	−0.39, −0.10	0.001	−0.15	−0.31, 0.01	0.067

	NHW vs. Black			NHW vs. Hispanic			Black vs. Hispanic		
**(C)**									
Ethnoracial group interaction	0.12	−0.01, 0.26	0.072	0.04	−0.10, 0.19	0.538	0.08	−0.03, 0.19	0.173

Standardized betas are shown. Sample sizes: total NHW cohort (*N* = 536), Aβ− NHW (*N* = 400), Aβ+ NHW (*N* = 136), total Hispanic cohort (*N* = 482), Aβ− Hispanic (*N* = 415), Aβ+ Hispanic (*N* = 67), total Black cohort (*N* = 546), Aβ− Black (*N* = 474), Aβ+ Black (*N* = 72).

Abbreviations: Aβ, amyloid‐β; CI, confidence interval; MTL, medial temporal lobe; NHW, non‐Hispanic White.

### 3.5 *APOE*ε4 did not moderate the associations between MTL tau and Aβ positivity in any of the ethnoracial groups

*APOE*ε4 positivity has been associated with greater tau PET SUVR signal in the MTL, independent of Aβ positivity status.^38^ However, prior studies have been performed in primarily NHW study samples and little is known about the associations between *APOE*ε4 positivity and MTL tau as well as the effect of *APOE*ε4 in the relationship between MTL tau and Aβ positivity in Hispanic and Black study samples.^23^ To evaluate the influence of *APOE*ε4 carriership on MTL tau levels across ethnoracial groups, we performed robust regression analyses controlling for age, sex, education, and Aβ positivity.

In the whole cohort, *APOE*ε4 positivity was associated with higher MTL tau levels (*β* = 0.12, 95% CI [0.02, 0.22], *p *= 0.023). The relationship between *APO*Eε4 and MTL tau was apparent in MCI participants (*β* = 0.32, 95% CI [0.09, 0.55], *p *= 0.007) but not CU participants (*β* = 0.05, 95% CI [−0.07, 0.16], *p *= 0.428). There was no interaction effect of ethnoracial group between NHW and Black participants (*β* = −0.20, 95% CI [−0.43, 0.03], *p *= 0.088), NHW and Hispanic participants (*β* = −0.07, 95% CI [−0.34, 0.20], *p *= 0.604) or Black and Hispanic participants (*β* = −0.14, 95% CI [−0.41, 0.13], *p *= 0.319) in the association between *APOE*ε4 genotype and MTL tau. Additionally, *APOE*ε4 did not moderate the associations between MTL tau and Aβ positivity in any of the ethnoracial groups (NHW (*β* = 0.27, 95%CI [−0.24, 0.77], *p *= 0.297), Hispanic (*β* = 0.60, 95%CI [−0.24, 1.45], *p *= 0.162), or Black (*β* = 0.24, 95%CI [−0.46, 0.95], *p *= 0.497)). Table 4 demonstrates these results.

**TABLE 4 alz71226-tbl-0004:** (A) *APOE*ε4 associations with MTL tau across ethnoracial groups and (B) interactions between ethnoracial group and *APOE*ε4 on MTL tau.

NHW CU and MCI			Hispanic CU and MCI			Black CU and MCI		
*β*	95% CI	*p*‐value	*β*	95% CI	*p*‐value	*β*	95% CI	*p*‐value
**(A)**								
0.18	0.006, 0.35	0.042	0.15	−0.07, 0.37	0.190	0.02	−0.14, 0.18	0.788

NHW vs. Black			NHW vs. Hispanic			Hispanic vs. Black		
*β*	95% CI	*p*‐value	*β*	95% CI	*p*‐value	*β*	95% CI	*p*‐value
**(B)**								
−0.20	−0.43, 0.03	0.088	−0.07	−0.34, 0.20	0.604	−0.14	−0.41, 0.13	0.319

Abbreviations: *APOE*ε4, apolipoprotein E allele; CI, confidence interval; CU, cognitively unimpaired; MCI, mild cognitively impaired; MTL, medial temporal lobe; NHW, non‐Hispanic White.

### 3.6 *APOE*ε4 did not moderate the associations between MTL tau and cognitive performance in any ethnoracial group

Prior[Table alz71226-tbl-0003], [Table alz71226-tbl-0004] studies have found mixed associations between *APOE*ε4 positivity and cognitive performance among ethnoracially diverse participants. For example, while *APOE*ε4 positivity has been consistently identified as a strong genetic risk factor for cognitive decline in NHW participants, others have found *APOE*ε4 positivity to have mixed or even potentially protective effects on cognition in Black and Hispanic participants.^16^, ^19^, ^20^, ^21^, ^22^, ^39^ To further assess the association between higher MTL tau levels and worse cognitive performance, we assessed the impact of *APOE*ε4 positivity on the relationship between cognitive performance and MTL tau within each ethnoracial group. We covaried for age, education, sex, and Aβ positivity.

Within the whole cohort, *APOE*ε4 positivity did not moderate the associations between MTL tau and memory composite performance (*β* = −0.09, 95% CI [−0.20,0.01], *p *= 0.089). Additionally, *APOE*ε4 did not moderate the association between memory composite and MTL tau in any of the ethnoracial groups (NHW (*β* = −0.07, 95%CI [−0.25, 0.10], *p *= 0.425), Hispanic (*β* = −0.13, 95% CI [−0.29, 0.03], *p *= 0.100), and Black (*β* = −0.13, 95%CI [−0.30, 0.04], *p *= 0.128)).

### 3.7 Hispanic participants demonstrated higher tau SUVR levels in lateral temporal regions than NHW and Black participants

According to the amyloid cascade hypothesis, tau deposition initiates in the transentorhinal cortex and moves outside of the MTL to the lateral temporal cortex, cingulate, frontal, and parietal regions^40^ after pathological amyloid levels have been reached. Considering that Black and Hispanic participants showed higher MTL tau levels than NHW participants in our sample, we evaluated whether ethnoracial group differences exist solely in the MTL or extend throughout other AD‐relevant regions in the brain. We investigated the associations between ethnoracial group and tau in lateral temporal regions (including the inferior temporal, middle temporal, fusiform, and parahippocampal gyrus) given that tau progression first moves to the nearby temporal neocortex. We also tested whether ethnoracial differences existed in more advanced regions of tau staging, including the posterior cingulate and lateral parietal ROIs (Figure S2).

Among the whole cohort, Hispanic participants showed higher levels of inferior temporal tau than NHW participants (*β*= 0.12, 95%CI [0.04, 0.20], FDR‐corrected *p *= 0.026) but no other ethnoracial differences existed between NHW and Hispanic participants across any of the other regions (0.02 < *β* < 0.09, FDR‐corrected *p* > 0.104) (Table S4). Hispanic participants also showed higher inferior temporal tau (*β* = 0.11, 95% CI [0.03,0.19], FDR‐corrected *p *= 0.018) and parahippocampal tau (*β* = 0.15, 95%CI [0.05, 0.26], FDR‐corrected *p *= 0.018) than Black participants. There were no differences in tau in any of the lateral temporal regions between Black and NHW participants (−0.003 < *β* < 0.08, FDR‐corrected *p* > 0.403) (Table S4). Given that ethnoracial differences in MTL tau were more pronounced in the CU group than the MCI group in our initial analyses, we evaluated tau SUVRs separately between CU and MCI groups. In CU participants, Hispanic participants showed significantly higher tau levels than CU NHW participants in the inferior temporal (*β* = 0.16, 95% CI [0.07, 0.25], FDR‐corrected *p *= 0.002), middle temporal (*β* = 0.13, 95% CI [0.03, 0.22], FDR‐corrected *p *= 0.027), and parahippocampal gyrus (*β* = 0.14, 95% CI [0.02, 0.26], FDR‐corrected *p *= 0.036) regions but not the fusiform (*β* = 0.10, 95% CI [−0.002,0.20], FDR‐corrected *p *= 0.083). CU Hispanic participants also showed significantly higher tau levels than CU Black participants in the inferior temporal (*β* = 0.13, 95% CI [0.04,0.23], FDR‐corrected *p *= 0.029) but no other regions outside the MTL showed any differences (0.01 < *β* < 0.15, FDR‐corrected *p* > 0.055) (Table S4). CU Hispanic participants showed comparable tau levels in the posterior cingulate (*β* = 0.07, 95% CI [−0.05, 0.19], FDR‐corrected *p *= 0.340) and lateral parietal (*β* = 0.06, 95% CI [−0.07,0.19], FDR‐corrected *p *= 0.381) relative to NHW participants. CU Black and NHW participants showed comparable levels of tau in all regions outside the MTL (−0.002 < *β* < 0.09, FDR‐corrected *p* > 0.335) (Table S4).

Since the amyloid cascade hypothesis proposes that tau outside the MTL appears after global pathological amyloid accumulation, we investigated whether Hispanic participants also showed higher tau levels than the other ethnoracial groups when Aβ negative. CU Hispanic Aβ negative participants showed significantly higher tau in lateral temporal regions (inferior temporal: *β* = 0.16, 95% CI [0.06, 0.25], FDR‐corrected *p *= 0.006, middle temporal: *β* = 0.11, 95% CI [0.01,0.21], FDR‐corrected *p *= 0.044, parahippocampal: *β* = 0.16, 95% CI [0.04, 0,29], FDR‐corrected *p *= 0.027, fusiform: *β* = 0.12, 95% CI [0.01, 0.23], FDR‐corrected *p *= 0.044)) than NHW participants but not in posterior cingulate (*β* = 0.07, 95%CI [−0.06, 0.20], FDR‐corrected *p *= 0.302) or lateral parietal regions (*β* = 0.07, 95%CI [−0.07, 0.21], FDR‐corrected *p *= 0.302). CU Hispanic Aβ negative participants also showed significantly higher tau in inferior temporal (*β* = 0.14, 95%CI [0.05,0.24], FDR‐corrected *p *= 0.018) and parahippocampal regions (*β* = 0.18, 95%CI [0.05, 0.30], FDR‐corrected *p *= 0.019) compared to Black participants but comparable levels in all other regions (0.03 < *β* < 0.09, FDR‐corrected *p* > 0.185) (Table S4). No differences were observed between CU Aβ negative Black and NHW participants in any regions (0.002 < *β* < 0.08, FDR‐corrected *p* > 0.602) (Table S4).

We also assessed ethnoracial differences in tau outside the MTL within the CU Aβ positive and MCI cohorts. Tau levels were similar in all advanced cortical areas between CU Aβ positive NHW participants and CU Aβ positive Hispanic (−0.17 < *β* < 0.18, FDR‐corrected *p* > 0.755), CU Aβ positive NHW participants and CU Aβ positive Black participants (−0.03 < *β* < 0.14, FDR‐corrected *p* > 0.630), and CU Aβ positive Hispanic and CU Aβ positive Black participants (−0.25 < *β* < 0.01, FDR‐corrected *p *= 0.968) (Table S4). Within the MCI cohort, Black (−0.25 < *β* <− 0.07, FDR‐corrected *p* > 0.326) and Hispanic (−0.26 < *β* < 0.07, FDR‐corrected *p* > 0.550) participants showed similar tau levels in all regions outside the MTL compared to MCI NHW participants (Table S4). Results across all groups are broken down in Table S4.

### 3.8 Meningeal off‐target binding contributed to higher lateral temporal tau in Hispanic participants

Given that the meninges can introduce off‐target binding into tau signal, we evaluated whether meningeal off‐target binding contributed to the ethnoracial tau differences observed in distinct lateral temporal regions. Using tau PET SUVRs derived from eroded lateral temporal segmentations, we assessed ethnoracial differences in the combined cohort as well as in the CU and CU Aβ negative cohorts, as these were the groups that originally exhibited significant findings. Within the whole cohort, all lateral temporal regions showed similar signal across ethnoracial groups (0.0004 < *β* < 0.09, FDR‐corrected *p* > 0.270). Similarly, lateral temporal tau showed no differences associated with ethnoracial group in the CU cohort (0.03 < *β* < 0.13, FDR‐corrected *p* > 0.063) or the CU Aβ− cohort (0.02 < *β* < 0.14, FDR‐corrected *p* > 0.080) indicating that the higher lateral temporal tau in Hispanic participants relative to NHW and Black participants was explained by higher meningeal off‐target binding in these regions. Table S5 contains these results.

### 3.9 Off‐target binding from the choroid plexus partially contributed to ethnoracial group MTL tau differences

Off‐target tau PET binding in the choroid plexus may contaminate signal in the neighboring hippocampus by inflating MTL tau signal. To evaluate whether off‐target signal explained ethnoracial differences in MTL tau PET levels, we evaluated off‐target tracer binding from the choroid plexus that may be included in measured hippocampal tau PET signal. Higher choroid plexus signal was strongly related to higher hippocampal tau signal (*β* = 0.58, 95% CI [0.53,0.62], *p* < 0.001) and higher overall MTL tau signal (*β* = 0.43, 95%CI [0.39,0.46], *p* < 0.001) in the whole cohort and separately within CU (hippocampal (*β* = 0.58, 95%CI [0.53, 0.64], *p* < 0.001), MTL (*β* = 0.42, 95%CI [0.38, 0,46], *p* < 0.001)) and MCI groups (hippocampal (*β* = 0.57, 95%CI [0.48, 0.67], *p* < 0.001), MTL (*β* = 0.44, 95%CI [0.35, 0.52], *p* < 0.001)). We found that Hispanic (*β* = 0.37, 95%CI [0.24, 0.49], *p* < 0.001) and Black (*β* = 0.39, 95%CI [0.29, 0.50], *p* < 0.001) participants showed higher choroid plexus signal than NHW participants. Black (*β* = 0.36, 95% CI [0.24, 0.48], *p* < 0.001) and Hispanic CU (*β* = 0.39, 95%CI [0.24,0.53], *p* < 0.001) participants showed higher choroid plexus signal than CU NHW participants. Similarly, MCI Hispanic (*β* = 0.31, 95% CI [0.05,0.57], *p *= 0.021) and Black (*β* = 0.45, 95% CI [0.22, 0.69], *p* < 0.001) participants showed higher choroid plexus signal than MCI NHW participants with Black MCI participants showing the strongest effect of choroid plexus off‐target signal of either group.

To evaluate whether choroid plexus off‐target binding affected our reported MTL tau associations, we tested the associations between ethnoracial group and MTL tau while controlling for choroid plexus SUVR. In the full sample, the association between MTL tau and ethnoracial group became non‐significant after controlling for choroid plexus signal such that Black participants no longer showed higher MTL tau levels than NHW participants (*β* = 0.08, 95%CI [−0.01,0.17], *p *= 0.072) while Hispanic participants still showed significantly higher MTL tau than NHW participants (*β* = 0.13, 95%CI [0.04, 0.22], *p *= 0.005) but with a reduced effect size relative to original findings. However, given that original associations between MTL tau and ethnoracial group differed between CU and MCI groups, we also evaluated MTL tau levels across ethnoracial groups separately in CU and MCI participants while adjusting for choroid plexus. In CU participants, we found that CU Hispanic (*β* = 0.13, 95%CI [0.04, 0.23], *p *= 0.006) and Black (*β* = 0.13, 95%CI [0.03, 0.23], *p *= 0.009) participants still showed higher MTL tau levels than CU NHW participants while controlling for choroid plexus. In those with MCI, Black (*β* = −0.12, 95%CI [−0.35, 0.11], *p *= 0.317) and Hispanic (*β* = 0.03, 95%CI [−0.22, 0.27], *p *= 0.819) participants still showed no significant difference in MTL tau levels compared to MCI NHW participants; however, relative to the original findings, the effect size was smaller, especially in the Black MCI participants which likely reduced the effect between Black and NHW participants in the overall combined group. After re‐performing the main analyses investigating the associations between MTL tau and Aβ positivity, cognitive performance, and *APOE*ε4 positivity while controlling for choroid plexus, we found that the results remained similar to original findings. Tables S6a–f contain these results.

Since it is likely that the choroid plexus affects MTL tau SUVR by increasing hippocampal tau signal, we tested the association between hippocampal tau and ethnoracial identity while controlling for choroid plexus. We found higher hippocampal tau levels among Black (*β* = 0.21, 95%CI [0.12, 0.30], *p* < 0.001) and Hispanic (*β* = 0.16, 95%CI [0.06,0.26], *p *= 0.002) participants relative to NHW participants, even after covarying for choroid plexus SUVR. To further understand the effect of hippocampal tau on MTL tau, we created a composite region that removed the hippocampus and solely consisted of the amygdala and entorhinal cortex. Assessing the relationships between ethnoracial group and the re‐created MTL composite demonstrated that Hispanic participants still showed higher MTL tau than NHW participants (*β* = 0.13, 95%CI [0.04, 0.22], *p *= 0.003); however, tau levels in Black participants were now similar to tau levels in NHW participants showing no significant differences (*β* = 0.02, 95%CI [−0.07,0.10], *p *= 0.721).

### 3.10 Ethnoracial group moderated the association between hippocampal tau SUVR and the learning and memory composite

In our lab's previous work, we found that using an MTL tau cut‐point differentiated NHW and Hispanic CU participants from cognitively impaired participants, but this was not the case for Black participants.^15^ However, in this previous study, the hippocampus was excluded from the MTL ROI, which influenced this ethnoracial difference in MTL tau associations with cognitive impairment. Since our current study's MTL ROI included the hippocampus and we have shown higher hippocampal tau levels in Black and Hispanic participants compared to NHW participants, we investigated whether hippocampal tau SUVRs significantly predicted cognitive performance across ethnoracial groups. All robust regressions controlled for age, sex, education, Aβ positivity, and choroid plexus PET signal. Higher hippocampal tau SUVR was associated with worse learning and memory composite score (*β* = −0.09, 95%CI [−0.15, −0.03], *p *= 0.002) in the overall CU and MCI cohort. Contrary to our MTL findings, ethnoracial group moderated the association between hippocampal tau SUVR and the learning and memory composite such that NHW participants showed an association between worse learning and memory scores and higher hippocampal tau SUVR whereas Black participants showed consistent learning and memory scores regardless of hippocampal tau SUVR level (*β* = 0.12, 95% CI [0.01, 0.23], *p *= 0.034). There was no interaction between Hispanic and NHW participants (*β* = 0.07, 95% CI [−0.05, 0.20], *p *= 0.249). Because we found that Aβ positivity moderated the association between MTL tau and cognitive performance in NHW and Hispanic cohorts but not in the Black cohort, we investigated whether the same was true of hippocampal tau. We found an interaction effect of Aβ positivity on the association between hippocampal tau and cognitive performance in both NHW participants (memory composite score (*β* = −0.33, 95% CI [−0.52, −0.14], *p* < 0.001)) and Hispanic participants (memory composite score (*β* = −0.23, 95% CI [−0.38, −0.07], *p *= 0.006) while Black participants showed no interaction effect of Aβ positivity (*β* = −0.12, 95% CI [−0.29, 0.05], *p *= 0.175).

## 4 DISCUSSION

We evaluated the associations between MTL tau, Aβ positivity, *APOE*ε4, and memory performance among ethnoracial groups of older adults without dementia. We found that Hispanic and Black participants had higher MTL than their NHW counterparts. Prior research evaluating MTL tau across ethnoracial groups^12^, ^13^ have reported mixed findings with one study finding no significant differences between Black and NHW participants. However, in that study, tau PET signal was measured in a composite ROI that included the occipital lobe^12^ which may have masked SUVR differences in early‐stage regions. Conversely, our past study found that, when no covariates were considered and the MTL composite excluded the hippocampus, Black participants demonstrated lower MTL tau SUVR levels than NHW and Hispanic participants.^15^ When we calculated the same unadjusted descriptive statistics using the MTL composite regions from our prior study (Supporting Information S3), we replicated the reported results (Table S7). Our current study includes the hippocampus in the MTL which we have identified as having high tau deposition in the Black and Hispanic participants relative to NHW participants. Our MTL tau findings are comparable to a prior study that found higher hippocampal flortaucipir signal in Black participants than NHW participants before controlling for choroid plexus signal spillover.^13^ Another study similarly found higher global flortaucipir signal in Black participants, but only included 12 Black participants.^14^ After performing a sensitivity analysis, we showed that choroid plexus off‐target binding may artificially inflate the MTL tau levels in the Hispanic and Black cohorts, likely by affecting hippocampal tau signal; however, even after controlling for off‐target binding, the whole Hispanic cohort and the CU Black cohort still demonstrated more MTL tau than their NWH counterparts.

We investigated whether ethnoracial group differences in the associations between MTL tau and Aβ positivity may be reflected in advanced tau regions. When evaluating the whole cohort, compared to NHW participants, Hispanic, but not Black, participants showed higher levels of inferior temporal tau. CU Hispanic participants likewise demonstrated higher lateral temporal tau levels, even in the absence of pathological amyloid, relative to CU Aβ− NHW and Black participants. However, after conducting a sensitivity analysis that re‐evaluated lateral temporal tau after removal of meningeal off‐target signal, we demonstrated that Hispanic lateral temporal tau levels were no different than Black and NHW lateral temporal tau levels. These results clarify that ethnoracial tau differences among older adults without dementia primarily reside in the MTL. Additionally, these findings show that PI2620 off‐target binding varies across ethnoracial groups with Hispanic participants demonstrating higher off‐target binding in the lateral temporal regions than Black and NHW participants. If not accounted for or removed, apparent tau levels for this ethnoracial group may be erroneously inflated. Only one study has found ethnoracial differences in choroid plexus off‐target binding between NHW and Black participants but this was observed using the 18F‐flortaucipir tracer.^13^ Researchers suggested that higher melanocyte levels may possibly contribute to these ethnoracial differences.^13^ Future research should investigate the underlying neurochemical or neuroanatomical drivers that increase susceptibility of off‐target PI2620 PET binding in certain ethnoracial groups over others. Additionally, researchers should account for this potential variability when developing study protocols and performing statistical analyses that include ethnoracially diverse older adults.

We found that ethnoracial identity did not moderate the association between MTL tau and cognitive performance. However, Aβ positivity moderated the association between higher MTL tau and lower memory scores in the Hispanic and NHW participants, but not in the Black participants. Our results suggest that in Black participants, factors other than amyloid and tau, such as vascular comorbidities, may have stronger associations with cognitive impairment and add variability, making the relationships between tau, amyloid, and cognitive performance harder to detect. Our previous work similarly showed that an MTL tau PET cut‐point acceptably distinguished cognitive impairment status only in the presence of Aβ but not in Black participants.^15^ Possibly this is because Black participants are more likely to develop dementia of a mixed or vascular etiology,^41^ which may be more strongly related to cognitive decline than AD biomarkers. Relative to NHW participants, Black participants had similar rates of cardiovascular disease including cardiomyopathy, atrial fibrillation, heart attack, heart failure, and valve replacement. Although previous literature has indicated that Black participants are more likely to experience cardiovascular disease compared to NHW participants,^42^ NHW participants in this study sample may have shown similar cardiovascular disease burden because they were on average 7–8 years older than the Black participants. However, Black participants showed significantly higher rates of hypertension and diabetes compared to NHW participants, which can promote adverse cerebrovascular co‐pathologies that could attenuate the relationship between tau, amyloid, and cognition due to the robust effects of vascular risk on cognitive performance. Exploration of cerebrovascular pathologies and their associations with tau and cognition were beyond the scope of this study. Future studies should assess how tau and amyloid interacts with other risk factors to affect cognition across ethnoracial groups.

We investigated how the hippocampus impacts the associations between MTL tau, cognitive performance, and ethnoracial group. Our regression results showed that when the hippocampus was excluded from the MTL composite, Hispanic participants showed higher MTL tau than NHW and Black participants suggesting that tau in Hispanic participants may be more evenly distributed throughout the MTL subregions than the Black participants who primarily demonstrate high MTL tau levels when the hippocampus is included. We also investigated whether the inclusion of hippocampal tau in the MTL composite would help explain associations with cognitive performance in the Black participants. Although Black participants had the highest hippocampal tau, regardless of choroid plexus off‐target binding, hippocampal tau was not associated with worse cognitive performance in the Black cohort, independent of Aβ positivity.

We investigated the potential moderating effect of *APOE*ε4 on the relationships between global Aβ and MTL tau, and MTL tau and memory performance, within the whole cohort and each ethnoracial group. *APOE*ε4 carriers had higher MTL tau than non‐carriers, independent of Aβ positivity. However, *APOE*ε4 carrier status did not moderate the association between MTL tau and Aβ positivity nor MTL tau and memory performance. Additionally, ethnoracial group showed no moderating effects in the association between *APOE*ε4 genotype and MTL tau suggesting similarities in the association between *APOE*ε4 and MTL tau levels across ethnoracial groups. In the future, more specific genetic markers such as the genetic variation local to the *APOE* region should be investigated as it relates to MTL tau associations with genotype. Black participants often have admixed ancestral heritage, containing both African and European ancestry, and thus have heterogenous ancestral background local to or near the *APOE* region.^43^ The effect of *APOE*ε4 on MTL tau may therefore be attenuated in Black participants with African ancestry local to the *APOE* region in a protective manner.^43^ Different from past studies, however, our study included a larger sample of Black participants with the statistical power to further explore moderation effects. Our analyses showed a similar effect of *APOE*ε4 carriership across ethnoracial groups in the relationship between Aβ and MTL tau as well as in the relationship between MTL tau and cognitive performance.

In conclusion, our findings suggest that in Black and Hispanic older adults without dementia, higher levels of MTL tau occur in the absence of pathological amyloid compared with NHW participants. The etiology of these higher tau levels is unclear but could be the consequence of a combination of genetic, health, and socioeconomic factors. Ethnically and racially minoritized groups are disproportionately burdened with acute and chronic exposure to stressors that cumulatively expedite the “weathering” or biological aging process^4^, ^5^, ^6^ and are associated with poorer episodic memory^5^ and greater brain aging.^7^ Transgenic rat models have shown that stress increases the hyperphosphorylation of phospho‐tau proteins.^44^, ^45^, ^46^ Stress‐related mechanisms may also be pathways for higher tau. Future research should evaluate the underlying reasons for higher MTL tau within Black and Hispanic cohorts.

There are a few limitations of our study. First, differences in the prevalence of health comorbidities, socioeconomic, cultural, and psychological stressors may be strong determinants of ethnoracial disparities in AD^4^, ^5^, ^6^, ^7^; however, we did not specifically interrogate how these factors may have contributed to the observed ethnoracial differences in tau distribution or the relationship between tau and cognition, an important topic for future work. Secondly, although we investigated MTL tau associations with *APOE*ε4 genotype, we did not evaluate how the genetic variation local to the *APOE* region is associated with MTL tau relationships. Future work should also consider cerebrovascular co‐pathologies such as infarcts or cerebral amyloid angiopathy that can co‐occur alongside tau biomarkers and lower the threshold for cognitive impairment. Particularly, Black older adults are at a greater risk of developing mixed AD and cerebrovascular pathologies than NHW participants^41^, ^47^ which can negatively impact episodic memory performance.^47^ Our study cohort contained a small sample of MCI participants across ethnoracial groups which restricted the statistical power to observe significant findings within this group. Lastly, our study was cross‐sectional and although we investigated tau levels across cognitive groups, we could not examine the trajectory of tau relationships with cognitive biomarkers over time like in a longitudinal study.

## CONFLICT OF INTEREST STATEMENT

The authors declare no conflicts of interest. Author disclosures are available in the Supporting Information.

## CONSENT STATEMENT

All participants in the HABS‐HD study provided informed consent, and the study protocol was approved by the Institutional Review Board at the University of North Texas Health Science Center (UNTHSC).

## Supporting information



Supporting Information

Supporting Information
