# Supplementary material for: The relationships between ethnoracial identity, Aβ positivity, APOEε4, and medial temporal lobe tau PET
Source: Alzheimers Dement. 2026 Mar 4;22(3):e71226. doi: 10.1002/alz.71226 (PMC12960054; doi:10.1002/alz.71226)
Supplement: Supplementary file 1 — Supporting Information [file ALZ-22-e71226-s002.docx]

**Supplementary Materials**

Supplemental Methods 1: Validation of amyloid positivity rates

Since the amyloid positivity rates of the study were at 18%, we tested that the ADNI3 amyloid positivity cutoff was the best fit for analyses in this cohort. We ran a Gaussian Mixture Model using the normalmixEM function from the mixtools package in R, the same statistical package that was used to derive the revised ADNI3 amyloid positivity cutoff of 1.08. We calculated the cutoff threshold using two methodologies. First, we used the methods performed by Drs. Jagust and Landau in the revised ADNI3 amyloid positivity cutoff methods. After the upper and lower distributions were fitted, the amyloid positivity cutoff was calculated as two standard deviations above the mean of the lower distribution resulting in a 1.072 cutoff value. In the second method, we calculated the point of intersection between the lower and upper distributions (the threshold at which the two components become separated) illustrated by **Supplemental Figure 1**. This cutoff value was determined to be 1.097. Given that the ADNI3 amyloid cutoff value of 1.08 falls in between the two cutoff values that we derived, we believe that the use of the ADNI3 cutoff value is appropriate for our cohort. Our amyloid positivity rate findings also align with several previous studies that have found lower amyloid positivity rates and lower continuous amyloid levels in study samples containing Black participants compared to predominantly NHW study samples^1^.

1. Deters, K. D., Napolioni, V., Sperling, R. A., Greicius, M. D., Mayeux, R., Hohman, T., & Mormino, E. C. Amyloid PET imaging in self-identified non-Hispanic Black participants of the anti-amyloid in asymptomatic Alzheimer's disease (A4) study. *Neurology*, 2021; *96*(11), e1491-e1500.

Supplemental Methods 2: Interpretation of MTL tau ethnoracial group differences using 1-to-1 matching

We performed a sensitivity analysis to test whether the MTL tau differences across ethnoracial groups originated from group characteristic differences related to disease progression. We matched the ethnoracial groups on study demographics of age (+/-3-year difference), gender, education in years, and cognitive diagnosis. We matched Black participants to NHW participants as well as Hispanic participants to NHW participants. Exact matches were found between Black-NHW and Hispanic-NHW pairs for gender, education in years, and cognitive diagnosis. To match pairs based on age, we used a cost matrix to match each Black or Hispanic participant to a NHW participant so that there was not an age difference of more than three years. Participants were only used once within each Black-NHW and Hispanic-NHW pair. The resulting sample sizes were 316 Black participants to 316 NHW participants and 201 Hispanic to 201 NHW participants. We re-performed analyses using these matched groups.

Overwhelmingly, results reflected similar findings to the main analyses. However, we suspect that due to the sample size reduction within the matched samples (42% reduction in Black participants and 58% reduction in Hispanic participants), some statistical power was lost so that only the strongest findings remained. Results from our matched samples showed that Black (β=0.31, 95% CI[0.20, 0.42], *p*<0.001) and Hispanic (β=0.27, 95%CI[0.15,0.40], *p*<0.001) participants demonstrated significantly higher MTL tau levels than NHW participants. Ethnoracial group did not significantly moderate the association between MTL tau and cognitive performance between any paired groups (-0.02<β<0.17, *p*>0.383). Aβ positivity moderated the association between MTL tau and learning and memory composite score across all ethnoracial groups (Black: β=-0.27, 95%CI[-0.46, -0.08], *p*=0.005, Hispanic: β=-0.39, 95%CI[-0.79, 0.0002], *p*=0.050 , NHW: β=-0.38, 95% CI[-0.55, -0.21], *p*<0.001), which differed from the original analyses in which Aβ positivity did not show a moderating effect in the Black group. Ethnoracial group did not moderate the association between MTL tau and *APOE*ε4 positivity (-0.15<β<-0.03, *p*>0.262). There was no effect of *APOE*ε4 in the association between MTL tau and Aβ positivity within the Hispanic (β=0.87, 95%CI[-0.05, 1.79], *p*=0.062), NHW (β=0.48 , 95%CI[-0.02, 0.98], *p*=0.062) or Black participants (β=-0.06, 95%CI[-0.78, 0.67], *p*=0.877). In our matched analyses, *APOE*ε4 continued to show no significant interaction in the association between MTL tau and cognitive performance among any of the ethnoracial groups (Black: β=-0.13, 95%CI [-0.35,0.09], *p*=0.242, NHW:β=-0.14, 95%CI[-0.49, 0.20], *p*=0.410, Hispanic: β=-0.27, 95%CI[-0.99,0.45], *p*=0.454). These results indicate that differences in group characteristics are not what is driving the associations between higher MTL tau among Hispanic and Black groups as most of our findings continued to show the same general strength and directionality even after significantly reducing the sample sizes from their original size.

Supplemental Methods 3: Assessment of Mean Tau Levels Using a MTL Composite of the Entorhinal Cortex, Parahippocampal Cortex, and Amygdala

To address the differences in MTL tau findings between the current study and our lab’s previous tau cutoffs study that found higher levels of MTL tau in NHW participants than Black and Hispanic participants, we calculated the mean tau levels of a MTL composite region which in the past study was comprised of the entorhinal cortex, parahippocampal cortex, and amygdala excluding the hippocampus. Mean MTL tau PET uptake was volume-weighted. To compare with our prior study’s supplemental findings^15^, we calculated means and standard deviations separately within each ethnoracial group (**Supplemental Table 6**). No adjustments were made for age, sex, education or Aβ positivity. Similar to our previous paper, we found that when using a MTL ROI consisting of the amygdala, parahippocampal and entorhinal cortex, NHW participants showed the highest MTL tau levels followed by Hispanic then Black participants. These results contrast with our current findings in which Black and Hispanic participants show MTL tau mean levels that are higher than NHW participants. However, as shown here, the difference in region of interest, use of descriptive statistics, and lack of covariates in our prior work explains the difference between study results. **Supplemental Table** **6** displays these results. It is worth noting that the NHW participants are on average older than the Black and Hispanic participants in HABS-HD, so greater tau accumulation in NHW participants is not surprising when age is not considered.

Supplemental Figure 1- Density Curves of Global Amyloid SUVRs


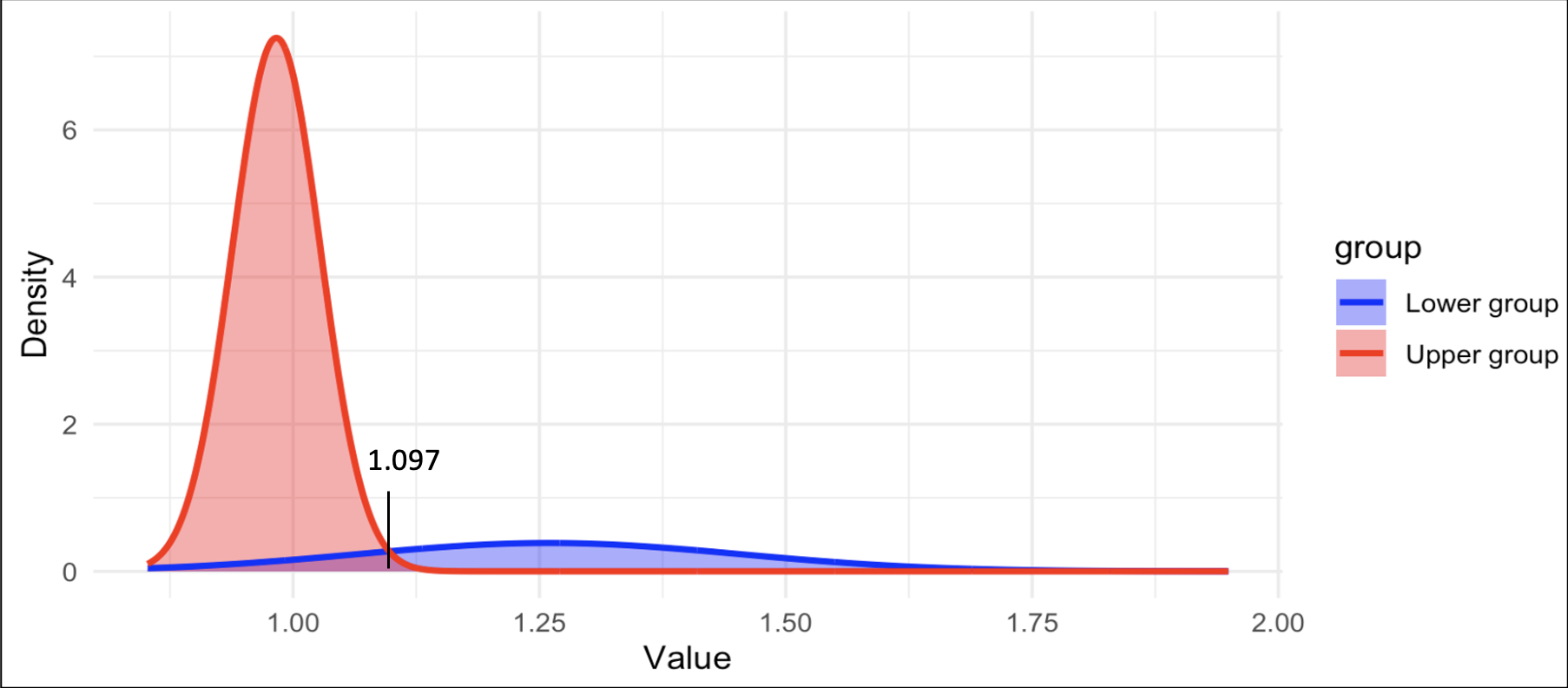


Supplemental Figure 2- Observed Posterior Cingulate and Lateral Parietal SUVRs across ethnoracial groups


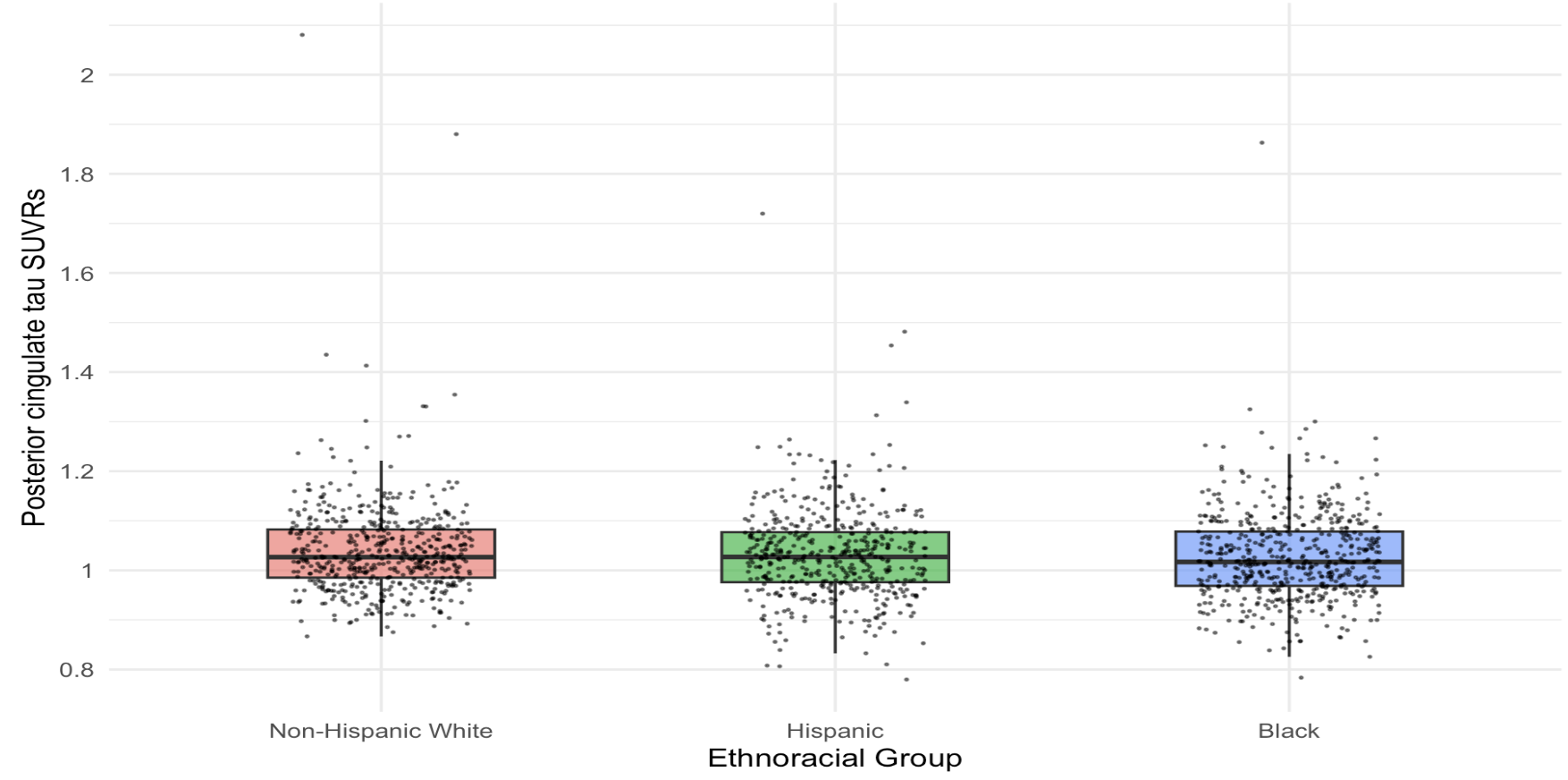


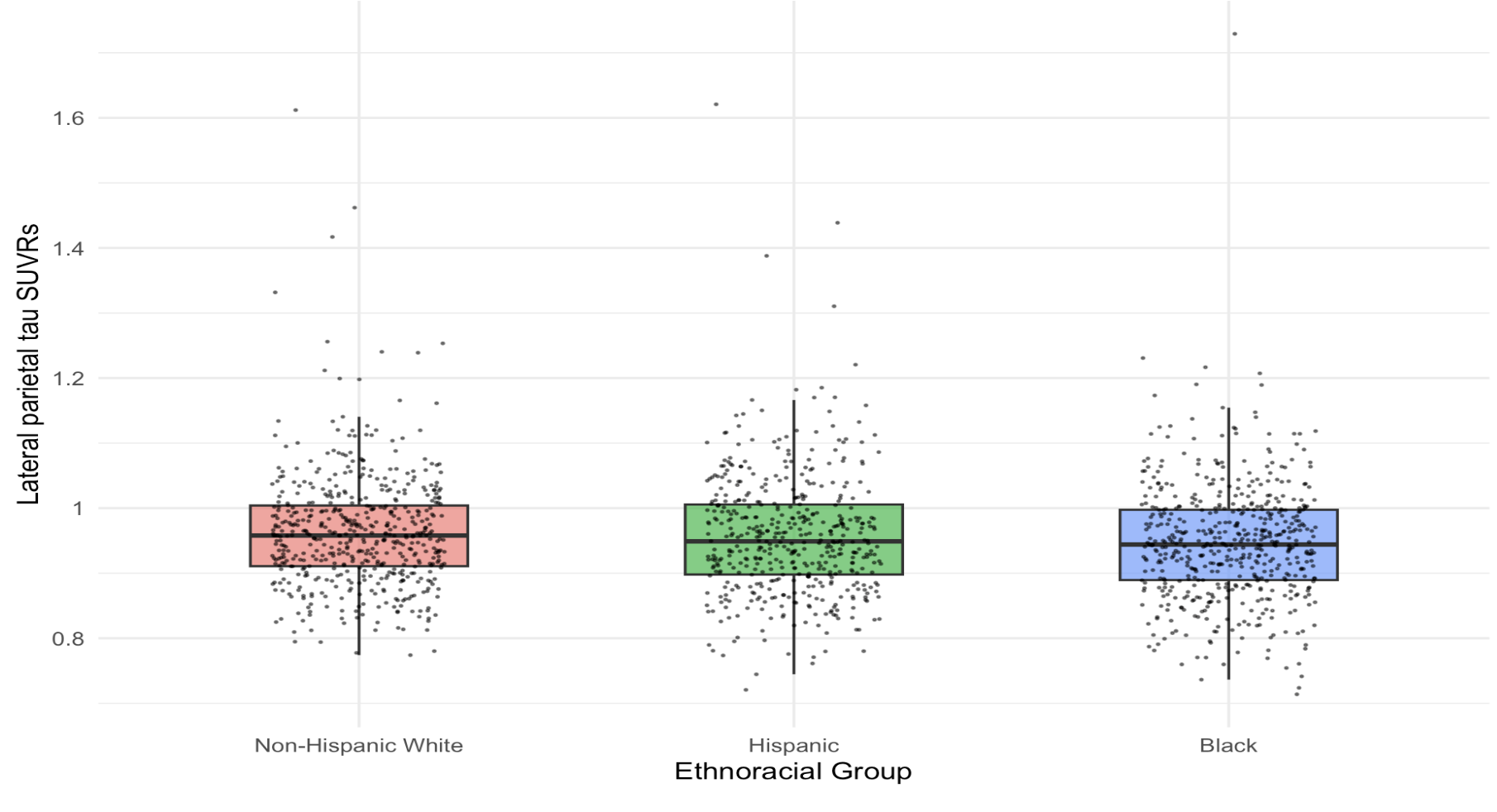


Boxplot depicts the distribution of posterior cingulate and lateral parietal tau SUVRs in all combined CU and MCI ethnoracial groups showing variability of posterior cingulate and lateral parietal tau in pre-clinical and early stages of cognitive impairment.

Supplemental Table 1. Robust regression results showing the associations between ethnicity and MTL tau SUVRs in CU and MCI participants

| Cognitive Diagnosis | β | 95% CI | *p* | β | 95% CI | *p* |
| --- | --- | --- | --- | --- | --- | --- |
|  | **Hispanic vs Non-Hispanic White participants** | | | **Black vs Non-Hispanic White participants** | | |
| Total CU and MCI cohort | 0.34 | 0.23, 0.44 | <0.001 | 0.28 | 0.19, 0.38 | <0.001 |
| Total CU cohort | 0.35 | 0.24, 0.46 | <0.001 | 0.32 | 0.21, 0.43 | <0.001 |
| Total MCI cohort | 0.21 | -0.08, 0.51 | 0.159 | 0.12 | -0.13, 0.38 | 0.354 |

In the full sample, we found that Hispanic and Black participants had higher MTL tau than NHW participants when controlling for age, sex, education, and Aβ positivity. To further investigate these significant results, we tested the associations between ethnoracial group and MTL across groups by cognitive diagnostic group. Standardized beta values are shown. Sample sizes: total CU cohort (N= 1181) and total MCI cohort (N=383).

Supplemental Table 2. Robust regression results showing interactions between ethnicity and Aβ on MTL tau SUVRs stratified by cognitive diagnosis.

|  | Cognitive Diagnosis | β | | 95% CI | | *p* | β | | 95% CI | *p* |
| --- | --- | --- | --- | --- | --- | --- | --- | --- | --- | --- |
|  |  | **Hispanic vs Non-Hispanic White participants** | | | | | **Black vs Non-Hispanic White participants** | | | |
| MTL tau associations | CU cohort | -0.13 | -0.54, 0.28 | | 0.530 | | 0.05 | -0.39, 0.49 | | 0.834 |
|  | MCI cohort | -0.53 | -1.40,  0.35 | | 0.239 | | -0.88 | -1.88,  0.12 | | 0.084 |

We performed formal interactions between Aβ positivity and ethnoracial group on MTL tau by cognitive status. Standardized beta values are shown. Sample sizes: CU cohort (N=1181), CU non-Hispanic White cohort (N= 451), CU Hispanic cohort (N= 353), CU Black cohort (N= 377), MCI cohort (N= 383), MCI non-Hispanic White cohort (N= 85), MCI Hispanic cohort (N= 129), MCI Black cohort (N= 169).

Supplemental Table 3. Robust regression results showing associations between cognitive test performance and MTL tau SUVRs across ethnoracial groups.

| **MTL tau associations** | **Non-Hispanic White participants** | | | **Hispanic participants** | | | | | **Black participants** | | |
| --- | --- | --- | --- | --- | --- | --- | --- | --- | --- | --- | --- |
|  | β | 95% CI | *p* | | β | | 95% CI | *p* | *β* | 95% CI | *p* |
| SEVLT Immediate | -0.16 | -0.32, -0.003 | 0.046 | | | -0.11 | -0.19,  -0.02 | 0.014 | -0.002 | -0.09, 0.08 | 0.969 |
| SEVLT Delayed | -0.13 | -0.30, 0.04 | 0.125 | | | -0.14 | -0.23,  -0.05 | 0.003 | -0.07 | -0.16, 0.03 | 0.162 |
| Logical Memory Immediate (A+B) | -0.11 | -0.19, -0.04 | 0.003 | | | -0.09 | -0.18, 0.005 | 0.063 | -0.03 | -0.12, 0.07 | 0.602 |
| Logical Memory Delayed (A+B) | -0.12 | -0.21, -0.03 | 0.012 | | | -0.12 | -0.23, 0.0003 | 0.051 | -0.07 | -0.16, 0.02 | 0.150 |

In the full sample, we found higher MTL tau was significantly associated with lower memory performance in NHW and Hispanic participants but not Black participants. To further explore these significant findings in the NHW and Hispanic groups, we examined the associations between MTL tau and each individual test that comprised the learning and memory composite. Standardized betas are shown. Sample sizes: non-Hispanic White (N= 536), Hispanic (N= 482), and Black (N=546) participants.

Supplemental Table 4. Robust regression results showing the associations between ethnicity and lateral parietal tau, posterior cingulate tau, and lateral temporal regions in CU and MCI participants stratified by cognitive status and Aβ positivity

|  | Cognitive Diagnosis | Aβ | β | 95% CI | *p* | β | 95% CI | *p* | β | 95% CI | *p* |
| --- | --- | --- | --- | --- | --- | --- | --- | --- | --- | --- | --- |
| Lateral Parietal |  |  | **Hispanic vs Non-Hispanic White participants** | | | **Black vs Non-Hispanic White participants** | | | **Hispanic vs Black participants** | | |
|  | **Total CU and MCI cohort** |  | 0.02 | -0.10, 0.14 | 0.760 | -0.003 | -0.11, 0.10 | 0.959 | 0.03 | -0.09, 0.15 | 0.908 |
|  | **Total CU cohort** | all | 0.06 | -0.07, 0.19 | 0.381 | 0.02 | -0.10, 0.13 | 0.894 | 0.04 | -0.09, 0.17 | 0.740 |
|  |  | Aβ- | 0.07 | -0.07, 0.21 | 0.302 | 0.01 | -0.12, 0.14 | 0.962 | 0.06 | -0.10, 0.22 | 0.546 |
|  |  | Aβ+ | -0.05 | -0.49, 0.38 | 0.933 | 0.04 | -0.21, 0.29 | 0.886 | -0.05 | -0.58, 0.47 | 0.968 |
|  | **Total MCI cohort** | all | -0.26 | -0.57, 0.04 | 0.550 | -0.25 | -0.50, 0.005 | 0.326 | -0.01 | -0.27, 0.25 | 0.976 |
|  |  | Aβ- | -0.18 | -0.49, 0.13 | 0.872 | -0.19 | -0.45, 0.06 | 0.527 | 0.03 | -0.24, 0.29 | 0.993 |
|  |  | Aβ+ | -0.69 | -1.69, 0.30 | 0.715 | -0.62 | -1.53, 0.29 | 0.990 | -0.21 | -1.15, 0.74 | 0.996 |
| Posterior cingulate | **Total CU and MCI cohort** |  | 0.03 | -0.08, 0.15 | 0.687 | 0.03 | -0.07, 0.13 | 0.912 | 0.02 | -0.10, 0.14 | 0.908 |
|  | **Total CU cohort** | all | 0.07 | -0.05, 0.19 | 0.340 | 0.04 | -0.08, 0.15 | 0.894 | 0.03 | -0.10, 0.17 | 0.740 |
|  |  | Aβ- | 0.07 | -0.06, 0.20 | 0.302 | 0.01 | -0.12, 0.13 | 0.962 | 0.06 | -0.10, 0.22 | 0.546 |
|  |  | Aβ+ | -0.02 | -0.42, 0.39 | 0.933 | 0.12 | -0.13, 0.37 | 0.630 | -0.06 | -0.53, 0.41 | 0.968 |
|  | **Total MCI cohort** | all | -0.16 | -0.46, 0.14 | 0.656 | -0.13 | -0.38, 0.12 | 0.523 | -0.004 | -0.26, 0.25 | 0.976 |
|  |  | Aβ- | -0.14 | -0.46, 0.17 | 0.872 | -0.16 | -0.42, 0.10 | 0.527 | 0.04 | -0.23, 0.30 | 0.993 |
|  |  | Aβ+ | -0.29 | -1.12, 0.54 | 0.731 | 0.005 | -0.83, 0.84 | 0.990 | -0.22 | -1.09, 0.64 | 0.996 |
| Fusiform | **Total CU and MCI cohort** |  | 0.07 | -0.02, 0.17 | 0.185 | 0.08 | -0.01, 0.17 | 0.403 | -0.005 | -0.10, 0.09 | 0.923 |
|  | **Total CU cohort** | all | 0.10 | -0.002, 0.20 | 0.083 | 0.09 | -0.002, 0.19 | 0.335 | 0.01 | -0.10, 0.12 | 0.865 |
|  |  | Aβ- | 0.12 | 0.01, 0.23 | 0.044 | 0.08 | -0.02, 0.18 | 0.602 | 0.03 | -0.08, 0.15 | 0.546 |
|  |  | Aβ+ | -0.10 | -0.39, 0.19 | 0.755 | 0.14 | -0.15, 0.44 | 0.630 | -0.25 | -0.67, 0.16 | 0.968 |
|  | **Total MCI cohort** | all | -0.11 | -0.36, 0.13 | 0.656 | -0.08 | -0.32, 0.17 | 0.540 | -0.04 | -0.22, 0.14 | 0.976 |
|  |  | Aβ- | -0.07 | -0.31, 0.17 | 0.872 | -0.01 | -0.24, 0.21 | 0.958 | -0.06 | -0.24, 0.12 | 0.993 |
|  |  | Aβ+ | 0.18 | -0.78, 1.15 | 0.745 | 0.26 | -0.97, 1.50 | 0.990 | -0.13 | -1.43, 1.17 | 0.996 |
| Inferior Temporal | **Total CU and MCI cohort** |  | 0.12 | 0.04, 0.20 | 0.026 | 0.004 | -0.07, 0.08 | 0.959 | 0.11 | 0.03,  0.19 | 0.018 |
|  | **Total CU cohort** | all | 0.16 | 0.07, 0.25 | 0.002 | 0.02 | -0.07, 0.10 | 0.894 | 0.13 | 0.04,  0.23 | 0.029 |
|  |  | Aβ- | 0.16 | 0.06, 0.25 | 0.006 | 0.002 | -0.09, 0.09 | 0.962 | 0.14 | 0.05,  0.24 | 0.018 |
|  |  | Aβ+ | 0.12 | -0.14, 0.38 | 0.755 | 0.11 | -0.15, 0.37 | 0.630 | 0.01 | -0.34, 0.35 | 0.968 |
|  | **Total MCI cohort** | all | -0.05 | -0.26, 0.15 | 0.656 | -0.13 | -0.33, 0.07 | 0.523 | 0.07 | -0.10, 0.24 | 0.976 |
|  |  | Aβ- | -0.07 | -0.27, 0.13 | 0.872 | -0.09 | -0.27, 0.09 | 0.527 | 0.03 | -0.14, 0.19 | 0.993 |
|  |  | Aβ+ | 0.38 | -0.43, 1.19 | 0.715 | 0.04 | -0.96, 1.04 | 0.990 | 0.15 | -1.06, 1.36 | 0.996 |
| Middle Temporal | **Total CU and MCI cohort** |  | 0.09 | -0.001, 0.18 | 0.104 | 0.03 | -0.06, 0.11 | 0.912 | 0.06 | -0.03, 0.14 | 0.368 |
|  | **Total CU cohort** | all | 0.13 | 0.03, 0.22 | 0.027 | 0.03 | -0.06, 0.12 | 0.894 | 0.09 | -0.01, 0.19 | 0.174 |
|  |  | Aβ- | 0.11 | 0.01, 0.21 | 0.044 | 0.01 | -0.09, 0.11 | 0.962 | 0.09 | -0.01, 0.19 | 0.185 |
|  |  | Aβ+ | 0.18 | -0.13, 0.48 | 0.755 | 0.11 | -0.15, 0.37 | 0.630 | 0.09 | -0.26, 0.43 | 0.968 |
|  | **Total MCI cohort** | all | -0.08 | -0.31, 0.16 | 0.656 | -0.07 | -0.29, 0.15 | 0.540 | -0.004 | -0.18, 0.18 | 0.976 |
|  |  | Aβ- | -0.001 | -0.25, 0.25 | 0.997 | 0.006 | -0.21, 0.22 | 0.958 | 0.001 | -0.18, 0.18 | 0.993 |
|  |  | Aβ+ | -0.14 | -0.98, 0.71 | 0.745 | -0.23 | -1.02, 0.57 | 0.990 | 0.003 | -1.23, 1.23 | 0.996 |
| Parahippocampal | **Total CU and MCI cohort** |  | 0.11 | 0.004,0.23 | 0.104 | -0.03 | -0.13, 0.07 | 0.912 | 0.15 | 0.05,  0.26 | 0.018 |
|  | **Total CU cohort** | all | 0.14 | 0.02, 0.26 | 0.036 | -0.002 | -0.11, 0.10 | 0.969 | 0.15 | 0.02,  0.27 | 0.055 |
|  |  | Aβ- | 0.16 | 0.04, 0.29 | 0.027 | -0.02 | -0.13, 0.09 | 0.962 | 0.18 | 0.05,  0.30 | 0.019 |
|  |  | Aβ+ | -0.17 | -0.56, 0.23 | 0.755 | -0.03 | -0.39, 0.34 | 0.886 | -0.13 | -0.55, 0.29 | 0.968 |
|  | **Total MCI cohort** | all | 0.07 | -0.24, 0.38 | 0.656 | -0.14 | -0.42, 0.15 | 0.523 | 0.21 | -0.02, 0.43 | 0.431 |
|  |  | Aβ- | 0.03 | -0.24, 0.30 | 0.995 | -0.11 | -0.35, 0.13 | 0.527 | 0.15 | -0.07, 0.36 | 0.993 |
|  |  | Aβ+ | 0.55 | -0.44, 1.55 | 0.715 | -0.10 | -1.18, 0.99 | 0.990 | 0.58 | -0.97, 2.14 | 0.996 |

We performed additional analyses to evaluate whether the associations with MTL tau across groups were also present in advanced tau regions of the lateral temporal, posterior cingulate, and lateral parietal. Standardized betas are shown. P values are FDR corrected. Sample sizes: total CU cohort (N= 1181), CU Aβ- (N=986), CU Aβ+ (N=195), total MCI cohort (N=383), MCI Aβ- (N= 303), MCI Aβ+ (N=80).

Supplemental Table 5. Sensitivity analysis results using robust regressions to test eroded lateral temporal tau SUVRs across ethnoracial groups

Table 5.

|  | Cognitive Status | Aβ | β | 95% CI | *p* | β | 95% CI | *p* | β | 95% CI | *p* |
| --- | --- | --- | --- | --- | --- | --- | --- | --- | --- | --- | --- |
| Fusiform |  |  | **Hispanic vs Non-Hispanic White participants** | | | **Black vs Non-Hispanic White participants** | | | **Hispanic vs Black participants** | | |
|  | **Total cohort** |  | 0.04 | -0.05, 0.13 | 0.0475 | 0.03 | -0.05, 0.12 | 0.811 | 0.01 | -0.08, 0.10 | 0.993 |
|  | **CU cohort** | all | 0.08 | -0.02, 0.18 | 0.121 | 0.05 | -0.04, 0.14 | 0.553 | 0.03 | -0.07, 0.13 | 0.571 |
|  |  | Aβ- | 0.09 | -0.01, 0.20 | 0.116 | 0.05 | -0.05, 0.15 | 0.712 | 0.04 | -0.07, 0.14 | 0.672 |
| Inferior Temporal | **Total cohort** |  | 0.07 | -0.02, 0.15 | 0.270 | -0.01 | -0.09, 0.07 | 0.811 | 0.07 | -0.01, 0.16 | 0.339 |
|  | **CU cohort** | all | 0.12 | 0.02, 0.21 | 0.063 | 0.03 | -0.06, 0.11 | 0.574 | 0.09 | -0.01, 0.18 | 0.371 |
|  |  | Aβ- | 0.11 | 0.004, 0.21 | 0.084 | 0.02 | -0.08, 0.11 | 0.712 | 0.08 | -0.03, 0.18 | 0.284 |
| Middle Temporal | **Total cohort** |  | 0.02 | -0.07, 0.11 | 0.618 | 0.02 | -0.06, 0.10 | 0.811 | 0.004 | -0.09, 0.09 | 0.993 |
|  | **CU cohort** | all | 0.08 | -0.02, 0.18 | 0.121 | 0.04 | -0.05, 0.13 | 0.553 | 0.03 | -0.07, 0.13 | 0.571 |
|  |  | Aβ- | 0.06 | -0.04, 0.17 | 0.239 | 0.03 | -0.07, 0.12 | 0.712 | 0.02 | -0.08, 0.13 | 0.684 |
| Parahippocampal | **Total cohort** |  | 0.09 | -0.02, 0.20 | 0.27 | 0.02 | -0.07, 0.12 | 0.811 | 0.07 | -0.03, 0.18 | 0.339 |
|  | **CU cohort** | all | 0.13 | 0.01, 0.24 | 0.068 | 0.06 | -0.05, 0.16 | 0.553 | 0.07 | -0.05, 0.19 | 0.538 |
|  |  | Aβ- | 0.14 | 0.02, 0.27 | 0.080 | 0.04 | -0.07, 0.15 | 0.712 | 0.10 | -0.03, 0.22 | 0.284 |

We performed additional analyses to evaluate whether the associations with lateral temporal tau across ethnoracial groups remained significant after an erosion method was used to remove potential meningeal off-target binding in this region. Standardized betas are shown. P values are FDR corrected. Sample sizes: total cohort (N=1564), total CU cohort (N= 1181), and CU Aβ- (N=986).

Supplemental Table 6. Robust regression results of MTL analysis including choroid plexus as a covariate

Table 6a. Interactions between ethnicity and Aβ on MTL tau SUVRs covarying for age, sex, education, and choroid plexus signal

|  | Cognitive Diagnosis | β | | 95% CI | | *p* | β | | 95% CI | *p* |
| --- | --- | --- | --- | --- | --- | --- | --- | --- | --- | --- |
|  |  | **Hispanic vs Non-Hispanic White participants** | | | | | **Black vs Non-Hispanic White participants** | | | |
| MTL tau associations | CU cohort | 0.07 | -0.27, 0.41 | | 0.677 | | 0.04 | -0.31, 0.38 | | 0.825 |
|  | MCI cohort | -0.54 | -1.28, 0.20 | | 0.151 | | -1.13 | -1.89,  -0.38 | | 0.003 |

Table 6b. Interactions between ethnicity and MTL tau SUVRs on memory performance covarying for age, sex, education, Aβ positivity, and choroid plexus signal

|  | β | | 95% CI | | *p* | β | | 95% CI | *p* | β | 95% CI | *p* |
| --- | --- | --- | --- | --- | --- | --- | --- | --- | --- | --- | --- | --- |
|  | **Hispanic vs Non-Hispanic White participants** | | | | | **Black vs Non-Hispanic White participants** | | | | **Hispanic vs Black participants** | | |
| MTL tau associations | 0.03 | -0.11, 0.18 | | 0.674 | | 0.11 | -0.02, 0.25 | | 0.108 | 0.08 | -0.03, 0.19 | 0.149 |

Table 6c. Interactions between Aβ positivity and MTL tau on cognitive performance across ethnoracial groups covarying for age, sex, education, and choroid plexus

|  | β | | 95% CI | | *p* | β | | 95% CI | *p* | β | 95% CI | *p* |
| --- | --- | --- | --- | --- | --- | --- | --- | --- | --- | --- | --- | --- |
|  | **NHW participants** | | | | | **Hispanic participants** | | | | **Black participants** | | |
| MTL tau associations | -0.30 | -0.41,  -0.18 | | <0.001 | | -0.22 | -0.37,  -0.07 | | 0.003 | -0.16 | -0.33, 0.004 | 0.055 |

Table 6d. Interactions between ethnicity and *APOE*ε4 genotype on MTL tau covarying for age, sex, education, Aβ positivity, and choroid plexus

|  | β | | 95% CI | | *p* | β | | 95% CI | *p* | β | 95% CI | *p* |
| --- | --- | --- | --- | --- | --- | --- | --- | --- | --- | --- | --- | --- |
|  | **Hispanic vs Non-Hispanic White participants** | | | | | **Black vs Non-Hispanic White participants** | | | | **Hispanic vs Black participants** | | |
| MTL tau associations | -0.04 | -0.26, 0.17 | | 0.696 | | -0.18 | -0.37, 0.02 | | 0.072 | -0.14 | -0.36, 0.09 | 0.230 |

Table 6e. Interactions between *APOE*ε4 positivity and Aβ positivity on MTL tau across ethnoracial groups covarying for age, sex, education, and choroid plexus

|  | β | | 95% CI | | *p* | β | | 95% CI | *p* | β | 95% CI | *p* |
| --- | --- | --- | --- | --- | --- | --- | --- | --- | --- | --- | --- | --- |
|  | **NHW participants** | | | | | **Hispanic participants** | | | | **Black participants** | | |
| MTL tau associations | 0.11 | -0.31, 0.54 | | 0.603 | | 0.63 | -0.29, 1.55 | | 0.180 | 0.15 | -0.34, 0.63 | 0.557 |

Table 6f. Interactions between *APOE*ε4 positivity and MTL tau on memory composite across ethnoracial groups covarying for age, sex, education, Aβ positivity, and choroid plexus

|  | β | | 95% CI | | *p* | β | | 95% CI | *p* | β | 95% CI | *p* |
| --- | --- | --- | --- | --- | --- | --- | --- | --- | --- | --- | --- | --- |
|  | **NHW participants** | | | | | **Hispanic participants** | | | | **Black participants** | | |
| MTL tau associations | -0.06 | -0.25, 0.12 | | 0.513 | | -0.12 | -0.28, 0.03 | | 0.122 | -0.13 | -0.31, 0.04 | 0.121 |

Supplemental Table 7. Means and SDs for MTL (a volume-weighted entorhinal, amygdala, and parahippocampal composite region) tau SUVRs between ethnoracial groups and by cognitive status without adjusting for covariates, methods mirroring our prior work^15^ for comparison purposes.

| Cognitive Diagnosis | Hispanic (N=482) | Black (N=546) | NHW (N=536) |  |  |
| --- | --- | --- | --- | --- | --- |
| Total CU cohort-mean (sd) | 1.140* (0.098) | 1.134* (0.114) | 1.168 (0.141) |  |  |
| Total MCI cohort-mean (sd) | 1.192* (0.187) | 1. 152* (0.166) | 1.257 (0.208) |  |  |
| Table of mean and standard deviations of MTL tau SUVRs created by a combination of volume-weighted entorhinal, amygdala, and parahippocampal regions across ethnoracial groups.  * indicates a statistically different MTL tau mean compared to the corresponding NHW group | | | | | |
|  | | | | | |
